# Hyperglycemic stress aggravates diabetic retinopathy and nephropathy by promoting cilium disassembly via a deacetylation- and methylation-mediated regulatory mechanism

**DOI:** 10.1371/journal.pbio.3003975

**Published:** 2026-09-03

**Authors:** Jie Ran, Changfeng Wei, Yang Yang, Yufei Zhang, Guizhi Guo, Nan Ma, Long Yin, Hongjun Fan, Jingrui Li, Heng Guo, Renshuai Zhang, Runa Wang, Dengwen Li, Min Liu

**Affiliations:** 1 Center for Cell Structure and Function, College of Life Sciences, Shandong Normal University, Jinan, China; 2 Translational Medicine Center, The First Affiliated Hospital of Zhengzhou University, Zhengzhou, China; 3 Department of Genetics and Cell Biology, State Key Laboratory of Medicinal Chemical Biology, College of Life Sciences, Nankai University, Tianjin, China; 4 School of Life Sciences and Medicine, Shandong University of Technology, Zibo, China; Rheinische Friedrich-Wilhelms-Universitat Bonn, GERMANY

## Abstract

Primary cilia are essential microtubule-based sensory organelles, and their dysfunction has been increasingly linked to metabolic stress. However, the underlying molecular mechanisms remain poorly understood. Herein, we reveal that ciliary defects in retinal photoreceptors and renal tubules exacerbate tissue damage during the progression of diabetic complications. Under hyperglycemic stress, protein arginine methyltransferase 1 (PRMT1) and histone deacetylase 6 (HDAC6) are significantly upregulated in both retinal and renal tissues. Genetic ablation of either enzyme effectively preserves ciliary architecture and restores organ function in diabetic mice. Mechanistically, PRMT1 localizes to the basal body, where it interacts with and methylates HDAC6 at arginine 16, consequently enhancing HDAC6 stability. In turn, HDAC6 mediates the deacetylation of PRMT1 at lysine 128, which elevates PRMT1 protein levels. This mutual modification crosstalk establishes a pathological positive feedback loop that stabilizes a pro-disassembly complex at the basal body, thereby potentiating ciliary impairment and expediting the progression of diabetic complications. Pharmacological inhibition of the PRMT1-HDAC6 loop significantly attenuates the pathological features of both diabetic retinopathy and nephropathy. Collectively, our findings uncover a reciprocal regulatory mechanism mediated by deacetylation and arginine methylation that drives cilium disassembly under hyperglycemic stress, providing promising therapeutic targets for the treatment of metabolic ciliopathies.

## Abstract 2

Primary cilium dysfunction contributes to diabetic complications, but the mechanisms linking hyperglycemia to cilium loss remain unclear. This study shows PRMT1-HDAC6 crosstalk drives cilium disassembly, identifying a therapeutic target for metabolic ciliopathies.

## Introduction

Primary cilia are microtubule-based sensory organelles that protrude from the surface of most mammalian cells, acting as critical hubs for integrating extracellular signals with intracellular response [1]. In recent years, the primary cilium has emerged as a central regulator of metabolic homeostasis, with structural or functional defects leading to a spectrum of disorders collectively termed “ciliopathies” [2,3]. These conditions often manifest with multi-organ impairments, including retinal degeneration, obesity, and renal dysfunction, which strikingly mirror the systemic complications observed in diabetic patients [4,5]. This parallel has led to the emerging concept of “metabolic ciliopathy”, suggesting that ciliary dysfunction may act as an important contributing factor that exacerbates diabetic complications, likely cooperating with established microvascular damage [6].

Diabetic retinopathy (DR) and diabetic nephropathy (DN) are the most prevalent and debilitating microvascular complications of diabetes, sharing common risk factors and a strong epidemiological link [7–9]. The “common soil” hypothesis posits that shared underlying mechanisms, such as chronic inflammation, oxidative stress, and endothelial dysfunction, exacerbate the progression of both conditions [10,11]. Recent studies have uncovered critical roles of cilia in diabetes-related conditions. For instance, in obesity, cilia on hypothalamic neurons regulate satiety signaling [3,12], while in pancreatic islets, cilia modulate insulin secretion through glucose-dependent mechanisms [13,14]. Moreover, ciliary defects impair osteogenesis in diabetic fracture healing and disrupt adipose tissue remodeling in response to dietary cues [15]. Despite these findings, the molecular links between ciliary dysfunction and diabetic complications, particularly in DR and DN, remain poorly understood.

The structural maintenance of primary cilium is dynamically governed by the balance between assembly and disassembly, a process exquisitely modulated by post-translational modifications (PTMs) [16]. PTMs of ciliary proteins significantly enhance the complexity of the ciliary proteome and are crucial for maintaining tissue integrity [17]. For instance, palmitoylation of ADP-ribosylation factor-like 13 B (ARL13B), a ciliary membrane-associated protein, is essential for protein trafficking and ciliary elongation, and its dysregulation has been implicated in atherosclerosis [18,19]. Additionally, our recent work reveals that UFMylation, a ubiquitin-like modification, promotes the stability of intraflagellar transport 88 (IFT88), maintaining ciliary homeostasis and multi-tissue integrity [20]. These findings underscore the importance of identifying novel ciliary proteins and their specific PTMs, which is critical for elucidating the regulatory mechanisms of ciliary homeostasis and developing targeted therapeutic strategies for ciliopathies. However, the regulatory mechanisms of ciliary proteins and the associated PTMs under metabolic stress remain largely unknown.

In this study, we identify a reciprocal regulatory circuit between protein arginine methyltransferase 1 (PRMT1)-mediated arginine methylation and histone deacetylase 6 (HDAC6)-mediated deacetylation that drives cilium disassembly in diabetic complications. Mechanistically, PRMT1 localizes to the ciliary basal body, where it interacts with and stabilizes HDAC6 through arginine 16 methylation. Reciprocally, HDAC6 enhances PRMT1 stability by mediating its deacetylation at lysine 128, creating a mutually reinforcing pathological loop. Our findings demonstrate that genetic or pharmacological disruption of this PRMT1-HDAC6 axis preserves ciliary integrity and ameliorates the structural and functional deficits associated with DR and DN. These results establish the PRMT1-HDAC6 feedback loop as a key molecular driver of metabolic ciliopathy, offering a promising therapeutic target for managing systemic diabetic complications.

## Results

### Hyperglycemic stress triggers cilium disassembly in retinal and renal tissues

**Fig 1:**
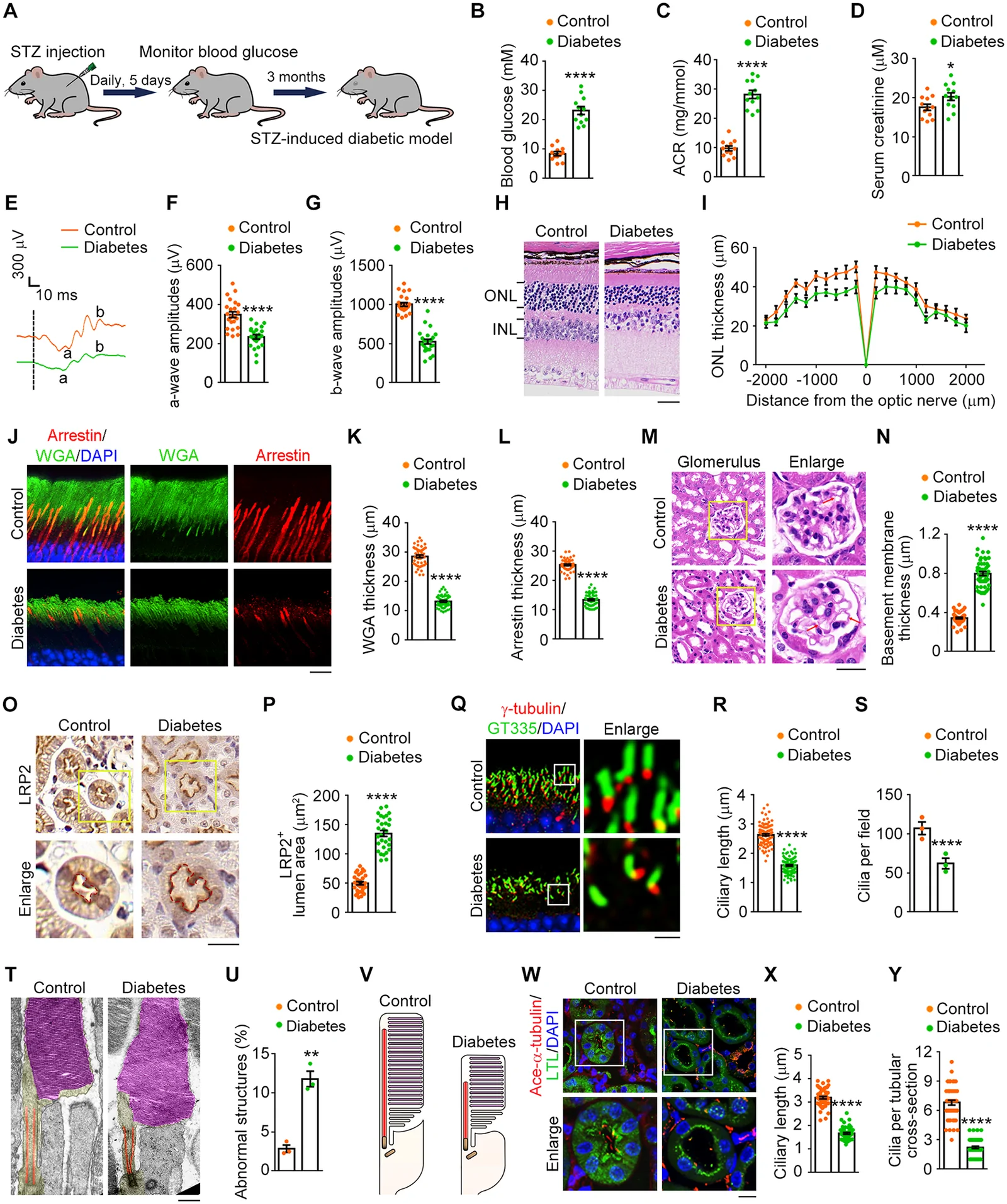
Impairment of retinal and renal structures under hyperglycemic stress. **(A)** A schematic diagram for the establishment of diabetic mouse model. Eight-week-old mice received daily intraperitoneal injections of streptozotocin (STZ, dissolved in 0.1 M citrate buffer) at the dose of 55 mg/kg, and mice received an equal volume of citrate buffer as controls. **(B–D)** Quantifications of blood glucose, albumin-to-creatinine ratio (ACR), and serum creatinine levels in diabetic and control mice (*n* = 12 mice from three independent experiments). **(E–G)** ERG recordings (E) and measurements of retinal a-wave (F) and b-wave (G) amplitudes were performed for the diabetic and control mice (*n* = 24 eyes from three independent experiments). **(H, I)** Photomicrographs (H) and quantification (I) of the retinal histology assessed by H&E staining in diabetic and control mice (*n* = 3 independent experiments). Scale bar, 40 μm. **(J–L)** Immunofluorescence images (J) and quantifications of membranous disc thickness in rods stained with WGA (K) and cones stained with the anti-cone arrestin antibody (L) from diabetic and control mice (*n* = 50 fields from three independent experiments). The maximum intensity projection of Z-stack images was used for quantification of the thickness of membranous discs of rods or cones with the ImageJ software. Scale bar, 10 μm. **(M, N)** Photomicrographs (M) and quantification of basement membrane thickness (N) from the renal histology assessed by H&E staining from diabetic and control mice (*n* = 50 fields from three independent experiments). Scale bar, 20 μm. **(O, P)** Photomicrographs (O) and quantification of the inner luminal area of proximal tubules (P) assessed by immunohistochemical staining for LRP2 from diabetic and control mice (*n* = 30 fields from three independent experiments). Dotted lines delineate the inner perimeters of LRP2^+^ tubules. Scale bar, 10 μm. **(Q–S)** Immunofluorescence images (Q) and quantifications of ciliary axoneme length (R, *n* = 100 fields from three independent experiments) and ciliary density (S, *n* = 3 independent experiments) in retinas stained with antibodies against polyglutamylated tubulin (GT335) and α-tubulin from diabetic and control mice. The maximum intensity projection of Z-stack images was used for quantification of axoneme length and ciliary density with the ImageJ software. Scale bar, 2 μm. **(T)** Transmission electron microscopy images of longitudinal sections of photoreceptors in diabetic and control mice. Scale bar, 1 μm. **(U)** Quantification of the percentage of abnormal structures in photoreceptor membranous discs from diabetic and control mice (*n* = 3 independent experiments using a total of 24 mice). **(V)** A schematic illustration of photoreceptors in control and diabetic mice. **(W–Y)** Immunofluorescence images (W) and quantifications of ciliary length (X) and density (Y) in kidney stained with antibody against acetylated α-tubulin (ace-α-tubulin), biotinylated Lotus tetragonolobus lectin (LTL), and DAPI in diabetic and control mice (*n* = 50 renal tubules from three independent experiments). The maximum intensity projection of Z-stack images was used for quantification of axoneme length and ciliary density with the ImageJ software. Scale bar, 6 μm. Data are presented as mean ± SEM. *p < 0.05, **p < 0.01, ****p < 0.0001. The data underlying this figure are available in S1 Data.

To investigate the pathophysiological processes underlying diabetic complications, we utilized a streptozotocin (STZ)-induced mouse model, a well-established approach for studying diabetes-related pathologies [21] (Fig 1A). Mice were subjected to a 5-day consecutive intraperitoneal injection of STZ at a dose of 55 mg/kg. Significant elevations in blood glucose levels were observed in diabetic mice, confirming the induction of persistent hyperglycemia (Fig 1B). Further analysis revealed a marked increase in the albumin-to-creatinine ratio in urine samples (Fig 1C), while the serum creatinine levels showed a mild elevation (Fig 1D), reflecting the early stage of renal involvement. Correspondingly, electroretinography (ERG) assessments demonstrated a remarkable reduction in both a-wave and b-wave amplitudes in diabetic mice, providing compelling evidence of visual dysfunction (Fig 1E–1G). These results confirm that STZ treatment effectively induces both renal and visual damage, thereby recapitulating key features of DN and DR in this model.

To further characterize the structural alterations associated with these functional impairments, we performed histological analyses of the retinal and renal tissues. Microscopic examination of hematoxylin and eosin (H&E)-stained retinal sections revealed a significant reduction in the thickness of the retinal outer nuclear layer (ONL) in diabetic mice compared to controls, with only mild thinning observed in the inner nuclear layer (Figs 1H, 1I, and S1A). This finding is consistent with the observed decline in ERG responses, as the ONL houses photoreceptor cell bodies, whose integrity is critical for normal visual function [22,23]. The thinning of the ONL in the diabetic mice aligns with established evidence of photoreceptor degeneration in STZ-induced models, further supporting the consistency of this pathological feature [24]. To investigate changes in rod and cone photoreceptors, particularly the membranous discs where phototransduction initiates [25], we stained these structures with wheat germ agglutinin (WGA) and anti-cone arrestin antibody to label rod and cone photoreceptors, respectively. Immunofluorescence microscopy revealed severe disruptions of the membranous discs of both rods and cones in diabetic mice (Fig 1J–1L), further corroborating the structural and functional deterioration of the retina. In parallel, histological analysis of the kidney revealed structural abnormalities in diabetic mice. H&E staining demonstrated thickening of the glomerular basement membrane and expansion of the mesangial matrix (Fig 1M and 1N). Additionally, segment-specific labeling revealed a pronounced dilation of the lumens in the proximal tubules, as indicated by low-density lipoprotein receptor-related protein 2 (LRP2) staining (Fig 1O and 1P). In contrast, the distal tubules labeled with Tamm–Horsfall protein (THP) exhibited no overt morphological alterations in their luminal area compared to controls (S1B and S1C Fig). This segment-specific pattern reflects the typical renal involvement characteristic of this early-stage model. These structural alterations align with the elevated ACR, highlighting the systemic impact of STZ-induced diabetes.

Given the critical role of cilia in maintaining tissue homeostasis, particularly in retinal photoreceptors for protein transport to membranous discs and in renal tubules for maintaining renal function [26,27], we next examined ciliary integrity in diabetic mice to determine whether ciliary disruption contributes to the observed structural and functional impairments. Immunostaining with antibodies targeting polyglutamylated tubulin (GT335), a well-known ciliary axoneme marker, and γ-tubulin, a centrosomal marker localized to the base of the cilium, showed that the length and density of the ciliary axoneme in retinal photoreceptors were significantly reduced in diabetic mice compared to controls (Fig 1Q–1S). To corroborate these observations, we performed transmission electron microscopy to examine both the longitudinal and cross sections of photoreceptors. Indeed, we found varying degrees of abnormalities in both the membranous discs and ciliary axonemes in the retinas of mice with DR (Figs 1T–1V and S1D). Furthermore, quantitative analysis of the cross-sections revealed a disorganized arrangement of microtubule doublets within the ciliary axonemes (S1E Fig). Similarly, cilia in the proximal tubules exhibited a marked reduction in both their length and density (Fig 1W–1Y), whereas those in the distal tubules remained largely unaffected (S1F–S1H Fig). Consistent with these ciliary defects in vivo, high-glucose treatment in cultured retinal RPE1 and renal IMCD3 cells directly triggered ciliary disassembly, resulting in a significant reduction in both ciliary length and density (S1I–S1K Fig), which confirms that hyperglycemic toxicity drives this process. These results collectively suggest that the disruption of cilia is a key cellular event associated with photoreceptor dysfunction and renal damage under hyperglycemic stress.

### Proteomic profiling identifies PRMT1 and HDAC6 as key drivers of diabetic ciliary defects

**Fig 2:**
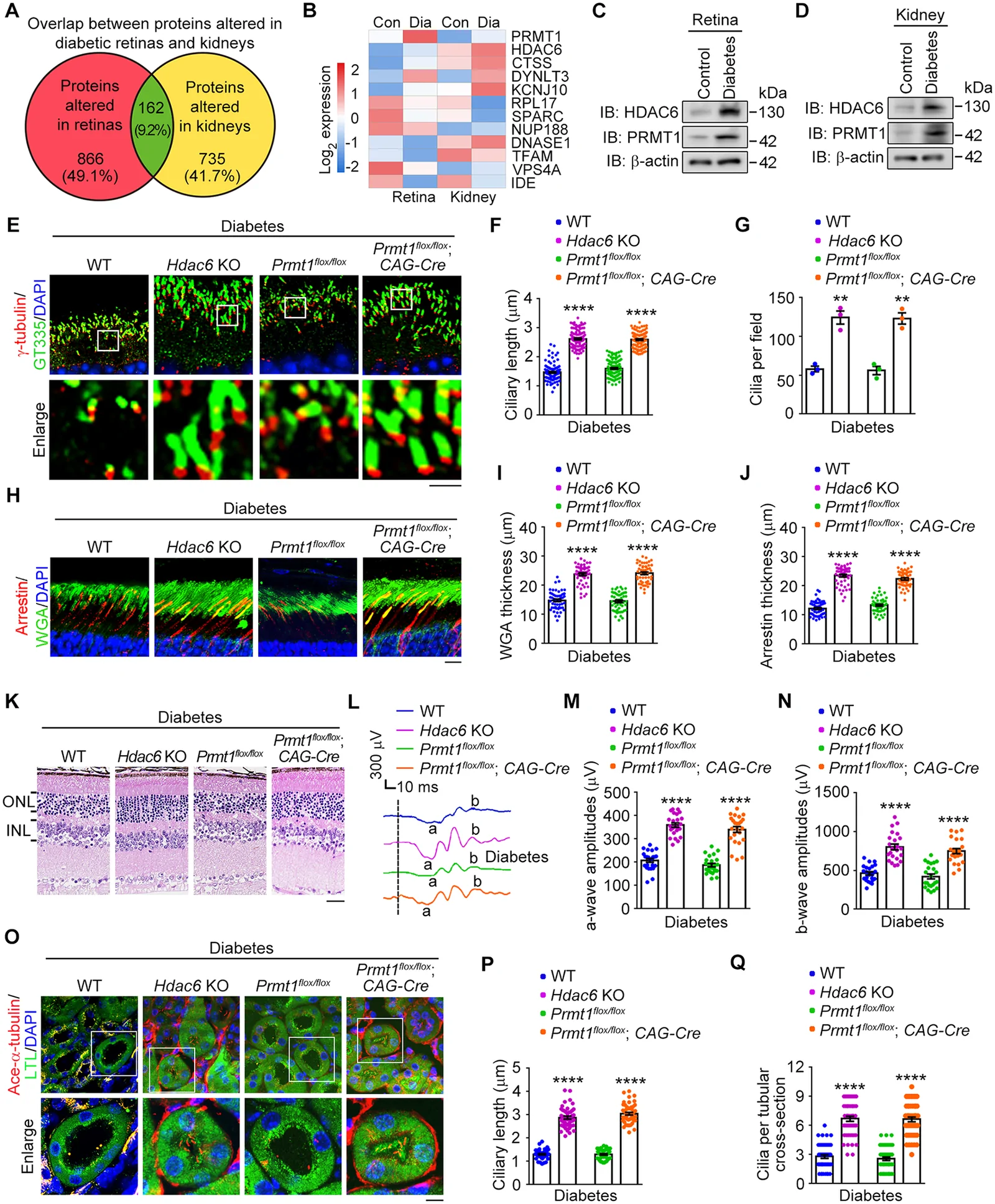
PRMT1 and HDAC6 overexpression exacerbates the progression of DR and DN. **(A)** Differentially expressed proteins in retinas and kidneys from control and diabetic mice. **(B)** Heat maps of representative cilium-associated proteins significantly altered in both retinas and kidneys from control and diabetic mice, filter criteria used DESeq *P*-values < 0.05, fold-change > 1.5. **(C, D)** Immunoblot analysis of HDAC6, PRMT1, and β-actin in the retina and kidney from control and diabetic mice. **(E–G)** Immunofluorescence images (E) and quantifications of the length (F, *n* = 100 fields from three independent experiments) and density (G, *n* = 3 independent experiments) of ciliary axonemes in retinas from *Hdac6* knockout or *Prmt1* conditional knockout mice and their respective controls under diabetic conditions. Scale bar, 2 μm. **(H–J)** Immunofluorescence images (H) and quantifications of the thickness of outer segment membranous disc in rods (I) and cones (J) from *Hdac6* knockout or *Prmt1* conditional knockout mice and their respective controls under diabetic conditions (*n* = 50 fields from three independent experiments). Scale bar, 10 μm. **(K)** Photomicrographs of the retinal histology assessed by H&E staining in *Hdac6* knockout or *Prmt1* conditional knockout mice and their respective controls under diabetic conditions. Scale bar, 30 μm. **(L–N)** ERG recordings (L) and measurements of retinal a-wave (M) and b-wave (N) amplitudes were performed for the *Hdac6* knockout or *Prmt1* conditional knockout mice and their respective controls under diabetic conditions (*n* = 24 mice from three independent experiments). **(O–Q)** Immunofluorescence images (O) and quantifications of ciliary length (P) and density in LTL^+^ proximal tubules from *Hdac6* knockout or *Prmt1* conditional knockout mice and their respective controls under diabetic conditions (*n* = 50 renal tubules from three independent experiments). Scale bar, 6 μm. Data are presented as mean ± SEM. **p < 0.01, ****p < 0.0001. The data underlying this figure are available in S1 Data and S1 Raw Images.

We then sought to investigate the molecular mechanisms underlying the pathological changes associated with DR and DN, including the damage to cilia in retinal photoreceptors and renal tubules. Mass spectrometry-based proteomic profiling was performed on STZ-treated retinas and kidneys, with untreated tissues serving as controls. Proteins exhibiting significant change (fold change ≥1.5, S1 Table) in both STZ-treated retinas and kidneys were selected for further investigation (Fig 2A). Among these, we identified a pronounced change of cilium-related proteins (Fig 2B). Specifically, we found that PRMT1, the most predominant type I PRMT responsible for asymmetric dimethylarginine (ADMA) formation [28], which plays a critical role in increasing ADMA levels in resorbing the flagella of *Chlamydomonas* [29,30]*,* and HDAC6, a unique cytoplasmic member of the HDAC family and a well-known cilium disassembly factor [31,32], were both significantly upregulated in diabetic retinas and kidneys (Fig 2B). Immunoblotting confirmed the increased levels of PRMT1 and HDAC6 in both STZ-treated tissues and high-glucose-treated IMCD3 cells compared to controls (Figs 2C, 2D and S2A). However, quantitative reverse transcription polymerase chain reaction (RT-qPCR) analysis revealed no significant differences in the mRNA levels of either *Prmt1* or *Hdac6* in the retinal and renal tissues between diabetic mice and controls (S2B and S2C Fig), indicating that the upregulation of PRMT1 and HDAC6 under hyperglycemic stress occurs at the post-translational level. These results suggest that the upregulation of PRMT1 and HDAC6 is closely associated with the pathological changes observed in DR and DN, implicating these proteins as potential therapeutic targets for these conditions.

To further validate their functional roles in the pathogenesis of DR and DN, we generated *Prmt1* (conditional, *Prmt1*^*flox/flox*^*; CAG-Cre-ERT2*) and *Hdac6* (systemic) knockout mice, and examined the structural and functional integrity of retinas and kidneys under diabetic conditions. Immunoblotting confirmed the absence of PRMT1 and HDAC6 in both retinas and kidneys derived from the respective knockout mice (S2D–S2G Fig). Immunofluorescence analysis demonstrated that depletion of PRMT1 or HDAC6 effectively hindered the impairment of ciliary axonemes and membranous discs associated with DR (Fig 2E–2J). Histopathological analysis of retinal morphology further demonstrated a significant rescue of photoreceptor ONL thickness upon PRMT1 or HDAC6 depletion (Figs 2K, S2H and S2I), indicating preservation of retinal structure. Consistent with these structural improvements, ERG analysis showed that the depletion of either PRMT1 or HDAC6 notably reversed reduction in both a-wave and b-wave amplitudes under diabetic conditions, reflecting the restoration of retinal function (Fig 2L–2N). These results suggest that depletion of PRMT1 or HDAC6 protects mice from DR-associated retinal deficits.

We next extended our investigation to the kidneys to evaluate the impact of PRMT1 and HDAC6 depletion on pathological changes associated with DN. Consistent with their preventive effects in the retina, depletion of PRMT1 or HDAC6 significantly ameliorated STZ-induced damages to glomerular and tubular structures (S2J–S2M Fig). Furthermore, the ciliary defects observed in proximal tubules under diabetic conditions were largely preserved in *Prmt1* or *Hdac6* knockout mice (Fig 2O–2Q). Importantly, these structural preservations were accompanied by significant improvements in renal function, as evidenced by a marked reduction of ACR in both knockout models (S2N Fig). Notably, in non-diabetic conditions, the depletion of PRMT1 or HDAC6 did not alter either baseline normoglycemia or the structure and function of the retina and kidney, aside from a marginal increase in ciliary incidence following *Prmt1* ablation (S2N, S3A–S3M, and S4A–S4G Figs). Furthermore, STZ-treated knockout mice developed profound hyperglycemia comparable to wild-type controls (S3A Fig), indicating that the genetic ablations are physiologically well-tolerated and do not prevent the systemic onset of diabetes, confirming that their protective effects arise specifically from counteracting the downstream pathological cascade driven by hyperglycemic stress. Collectively, these findings demonstrate that the upregulation of PRMT1 and HDAC6 promotes cilium disassembly, which is closely associated with the progression of the subsequent pathological changes observed in DR and DN.

### PRMT1 and HDAC6 form a sensory-localized complex at the ciliary basal body

**Fig 3:**
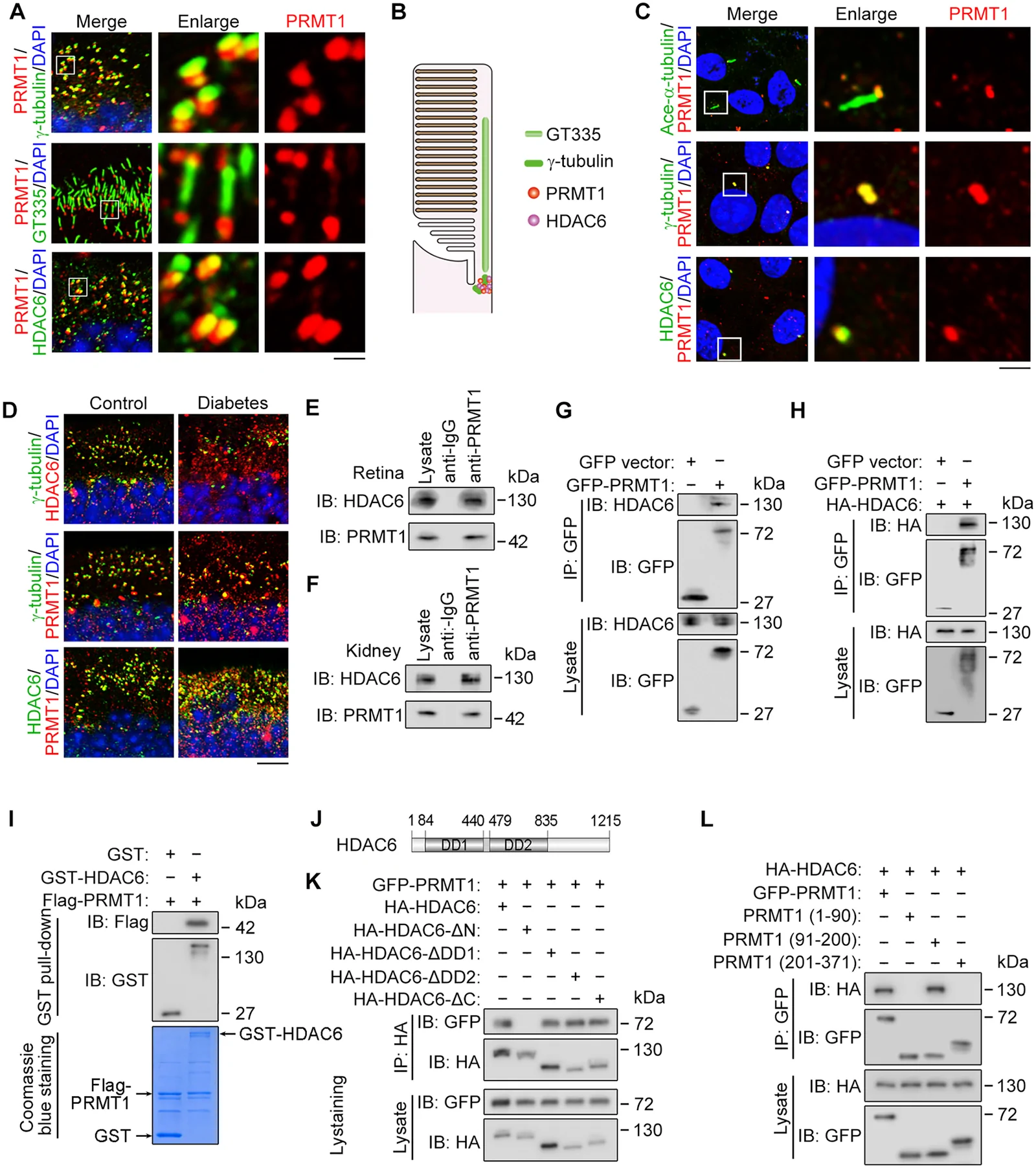
PRMT1 localizes to the basal body and interacts with HDAC6. **(A)** Immunofluorescence staining of retinal photoreceptors with antibodies against PRMT1, γ-tubulin, GT335, and HDAC6. Scale bar, 2 μm. **(B)** Graphic illustration of the co-localization of PRMT1 and HDAC6 in the retinal photoreceptors. **(C)** Immunofluorescence images of IMCD3 cells cultured in serum-free medium, and stained with antibodies against PRMT1 and α-tubulin, ace-α-tubulin, and HDAC6. Scale bar, 2 µm. **(D)** Immunofluorescence staining of retinal photoreceptors from control and diabetic mice with antibodies against PRMT1, HDAC6, and α-tubulin. Scale bar, 10 μm. **(E, F)** Immunoprecipitation and immunoblotting showing the interaction between endogenous PRMT1 and HDAC6 in the retina (E) and kidney (F) of mice. **(G, H)** Immunoprecipitation and immunoblotting showing the interaction of GFP-PRMT1 with endogenous HDAC6 (G) or HA-HDAC6 (H) in HEK293T cells. **(I)** GST pull-down assay showing the interaction of purified GST-HDAC6 with purified Flag-PRMT1 protein. **(J–L)** Identification of the domains mediating the interaction between PRMT1 and HDAC6 using various deletion constructs of HA-HDAC6 (K) or GFP-PRMT1 **(L)**, along with a schematic diagram of HDAC6 **(J)**. DD, deacetylase domain. The data underlying this figure are available in S1 Raw Images.

To elucidate the molecular mechanisms by which PRMT1 and HDAC6 contribute to the pathogenesis of DR and DN, we investigated the subcellular localization of these two proteins. Immunostaining results revealed that PRMT1 localizes to the centrosome of retinal photoreceptors, as evidenced by its co-localization with γ-tubulin (Fig 3A). This specific localization was confirmed by the complete absence of centrosomal signals in *Prmt1* knockout retinas (S5A Fig). Further co-staining with GT335 confirmed that PRMT1 specifically localizes to the ciliary basal body, a critical site for ciliary assembly and function (Fig 3A). Given that HDAC6 is a well-established centrosomal ciliary protein [33], we examined whether PRMT1 and HDAC6 exhibit co-localization. Immunofluorescence microscopy demonstrated that PRMT1 and HDAC6 co-localize at the ciliary basal body in retinal photoreceptors (Fig 3A and 3B). This co-localization was also observed in IMCD3 cells, further supporting the interaction between these two proteins (Fig 3C). To determine whether the localization of PRMT1 and HDAC6 is altered under diabetic conditions, we performed immunofluorescence microscopy, which revealed that both proteins remained detectable at the basal body, and the intensity of these two proteins was elevated in diabetic retinas compared to normal conditions (Figs 3D, S5B, and S5C).

Subsequent immunoprecipitation experiments confirmed the interaction between PRMT1 and HDAC6 in both retinal and renal tissues (Figs 3E, 3F, S5D, and S5E). This interaction was also observed in IMCD3 cells (S5F and S5G Fig). In addition, immunoprecipitation revealed that GFP-PRMT1 interacted with both endogenous HDAC6 and exogeneous HA-HDAC6, while GFP-HDAC6 interacted with Flag-PRMT1 in HEK293T cells (Figs 3G, 3H, and S5H). To further validate these findings, we performed GST pull-down assays using recombinant GST-HDAC6 and Flag-PRMT1 proteins, which confirmed the direct interaction between HDAC6 and PRMT1 in vitro (Fig 3I). To map the specific regions responsible for this interaction, we generated a series of truncated constructs of PRMT1 and HDAC6. Domain mapping analysis revealed that the amino-terminal region of HDAC6 is essential for its interaction with PRMT1, while the 91–200 amino acid (aa) region of PRMT1 is critical for its binding to HDAC6 (Fig 3J–3L). Collectively, these results demonstrate that PRMT1 and HDAC6 forms a stable functional complex at the ciliary base, providing a molecular basis for their potential functional partnership in regulating ciliary integrity and function under diabetic conditions.

### PRMT1-mediated arginine methylation stabilizes HDAC6 to promote ciliary resorption under diabetic conditions

**Fig 4:**
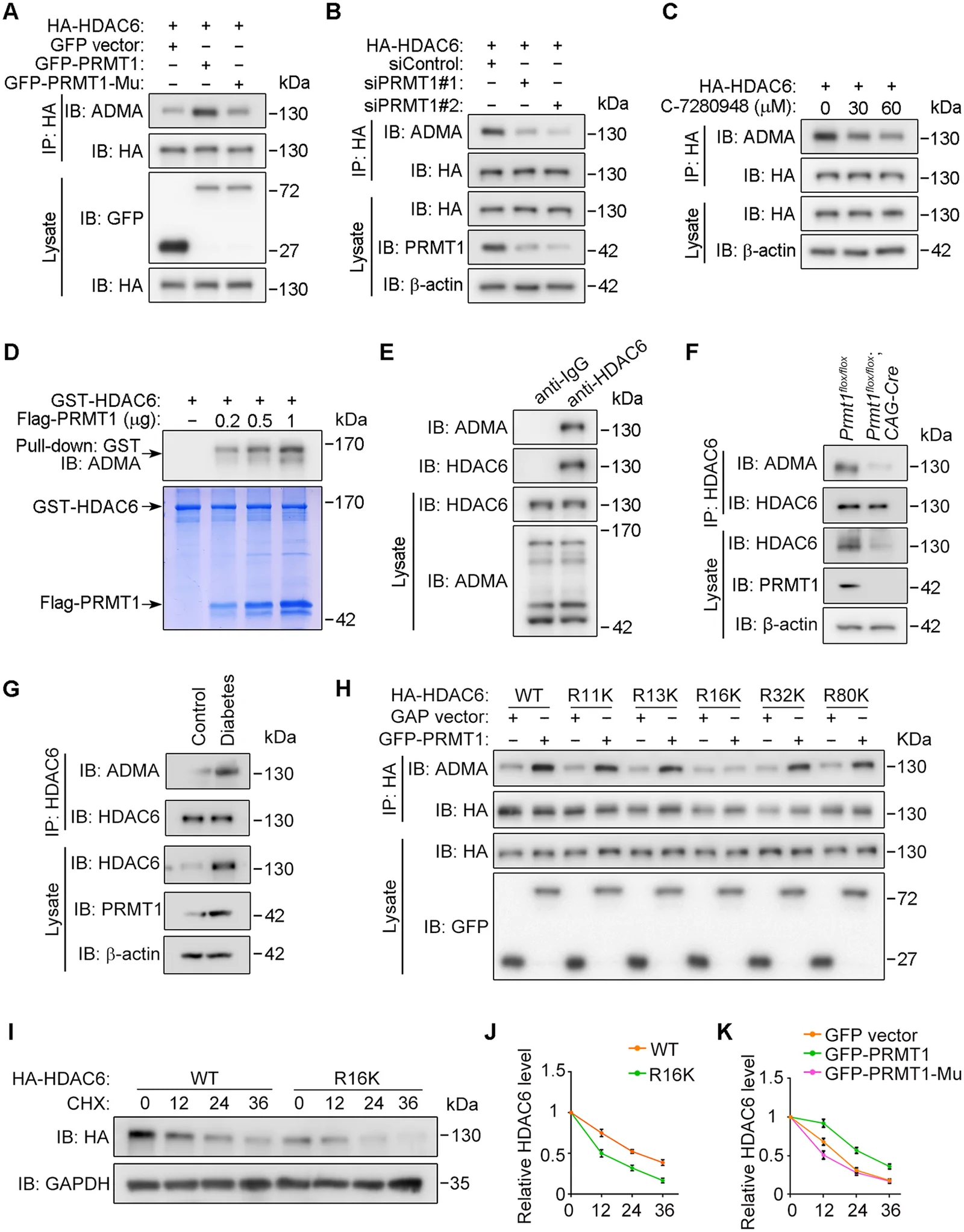
PRMT1-mediated asymmetric arginine dimethylation promotes HDAC6 stability. **(A)** The asymmetric demethylation of HDAC6 was analyzed by immunoprecipitation with the anti-HA antibody followed by immunoblotting in cells transfected with GFP-PRMT1, GFP-PRMT1 mutant (VLD-AAA, catalytically inactive mutant) or GFP vector, in the presence of HA-HDAC6. **(B, C)** Immunoprecipitation and immunoblotting analysis of asymmetric dimethylation of HDAC6 in cells transfected with control or PRMT1 siRNAs in the presence of HA-HDAC6 **(B)**, and in cells transfected with HA-HDAC6 with or without C-7280948 **(C)**. **(D)** In vitro HDAC6 dimethylation assay using purified GST-HDAC6 and various concentrations of Flag-PRMT1 as indicated. **(E)** Immunoprecipitation and immunoblotting analysis asymmetric dimethylation of endogenous HDAC6 in mouse retinas. **(F, G)** Analysis of the asymmetric dimethylation of endogenous HDAC6 in retinas from wild-type and *Prmt1* conditional knockout mice **(F)**, or from control and diabetic mice **(G)**. **(H)** Identification of the site of HDAC6 asymmetric dimethylation by immunoprecipitation with the anti-HA antibody followed by immunoblotting with the anti-ADMA antibody in cells transfected with GFP-PRMT1 or GFP vector in the presence of HDAC6 wild-type or arginine-to-lysine mutants. **(I, J)** The protein stability of wild-type or R16K mutant HDAC6 was examined by immunoblotting in cells transfected with HA-HDAC6 wild-type or the R16K mutant and treated with 20 μg/mL cycloheximide (CHX) for the indicated time. **(K)** Quantification of HDAC6 stability in cells transfected with GFP vector, GFP-PRMT1 or its mutant. The data underlying this figure are available in S1 Data and S1 Raw Images.

As the most predominant type I PRMT in mammalian cells, PRMT1 deposits an ADMA mark onto substrates and has been implicated in diverse cellular activities [28]. We next sought to explore the molecular mechanisms by which PRMT1 contributes to diabetic complications. We observed simultaneous elevation of PRMT1 and HDAC6 levels under diabetic conditions, suggesting a potential regulatory relationship between these two proteins. Moreover, PRMT1 interacts with HDAC6 at the ciliary basal body, and depletion of PRMT1 or HDAC6 could alleviate pathological changes, including ciliary defects associated with diabetic complications. These findings promoted us to speculate that PRMT1 might participate in diabetic complications through regulating the arginine methylation of HDAC6. To test this possibility, we transfected HA-HDAC6 and GFP-PRMT1 wild-type or its catalytically inactive mutant (V84-L85-D86 to A-A-A) to HEK293T cells. We found that the methylation of HDAC6 was rapidly increased in the presence of wild-type PRMT1, but not the catalytically inactive mutant (Fig 4A). Consistently, knockdown of PRMT1 with two different siRNAs or inhibition of PRMT1 with C-7280948, a selective PRMT1 inhibitor, efficiently decreased the methylation of HDAC6 (Fig 4B and 4C). We also performed in vitro methylation assays with recombinant GST-HDAC6 and varying concentrations of Flag-PRMT1, and observed an enhancement of HDAC6 methylation with increasing concentrations of PRMT1 protein (Fig 4D). Additionally, the methylation of endogenous HDAC6 was observed in the mouse retinal and renal lysates (Figs 4E and S6A), which was correspondingly downregulated upon PRMT1 depletion (Figs 4F and S6B). Notably, we detected a remarkable elevation of HDAC6 methylation in diabetic mice, compared with controls (Figs 4G and S6C), which was consistent with the observed upregulation of PRMT1 in these mice. Taken together, these results indicate that enhanced PRMT1 expression promotes the arginine methylation of HDAC6 under diabetic conditions.

To identify the methylated residues in HDAC6, the five arginines in the amino-terminal region of HDAC6 were mutated individually into lysines. Of these arginine-to-lysine (RK) mutations, only the R16K mutation restrained the methylation of HDAC6, indicating the occurrence of HDAC6 methylation mainly at the R16 residue (Fig 4H). In STZ-treated diabetic mice, we observed an increase in HDAC6 protein levels without significant changes in its subcellular localization (Fig 3D), prompting us to investigate whether PRMT1 regulates HDAC6 protein level. Indeed, depletion of PRMT1 led to a significant reduction in HDAC6 protein levels in both retinal and renal tissues (Figs 4F and S6B). Conversely, overexpression of wild-type PRMT1, but not its catalytically inactive mutant, significantly increased HDAC6 protein levels without affecting its mRNA expression (S6D–S6F Fig). These results suggest that HDAC6 methylation may influence its protein stability. To test this potential, we treated cells with the protein synthesis inhibitor cycloheximide and measured the half-life of HDAC6. The R16K mutant exhibited a much shorter half-life compared to wild-type HDAC6 (Fig 4I and 4J). Conversely, the half-life of HDAC6 was significantly prolonged in cells overexpressing wild-type PRMT1 but remained unchanged in cells expressing the catalytically inactive mutant (Figs 4K and S6G). Consistent with these findings, the less stable R16K mutant exhibited a markedly attenuated capacity to induce ciliary disassembly compared to wild-type HDAC6 in IMCD3 cells (S6H–S6J Fig). Furthermore, pharmacological inhibition of PRMT1 with C-7280948 significantly delayed the rate of serum-induced ciliary disassembly, indicating that these ciliary defects arise primarily from enhanced disassembly (S6K–S6M Fig). Collectively, these results indicate that increased PRMT1 promotes HDAC6 methylation at R16 to increase its stability, thereby elevating its capacity to trigger cilium disassembly under diabetic conditions.

### HDAC6 and PRMT1 constitute a positive-feedback regulatory loop in diabetic complications

**Fig 5:**
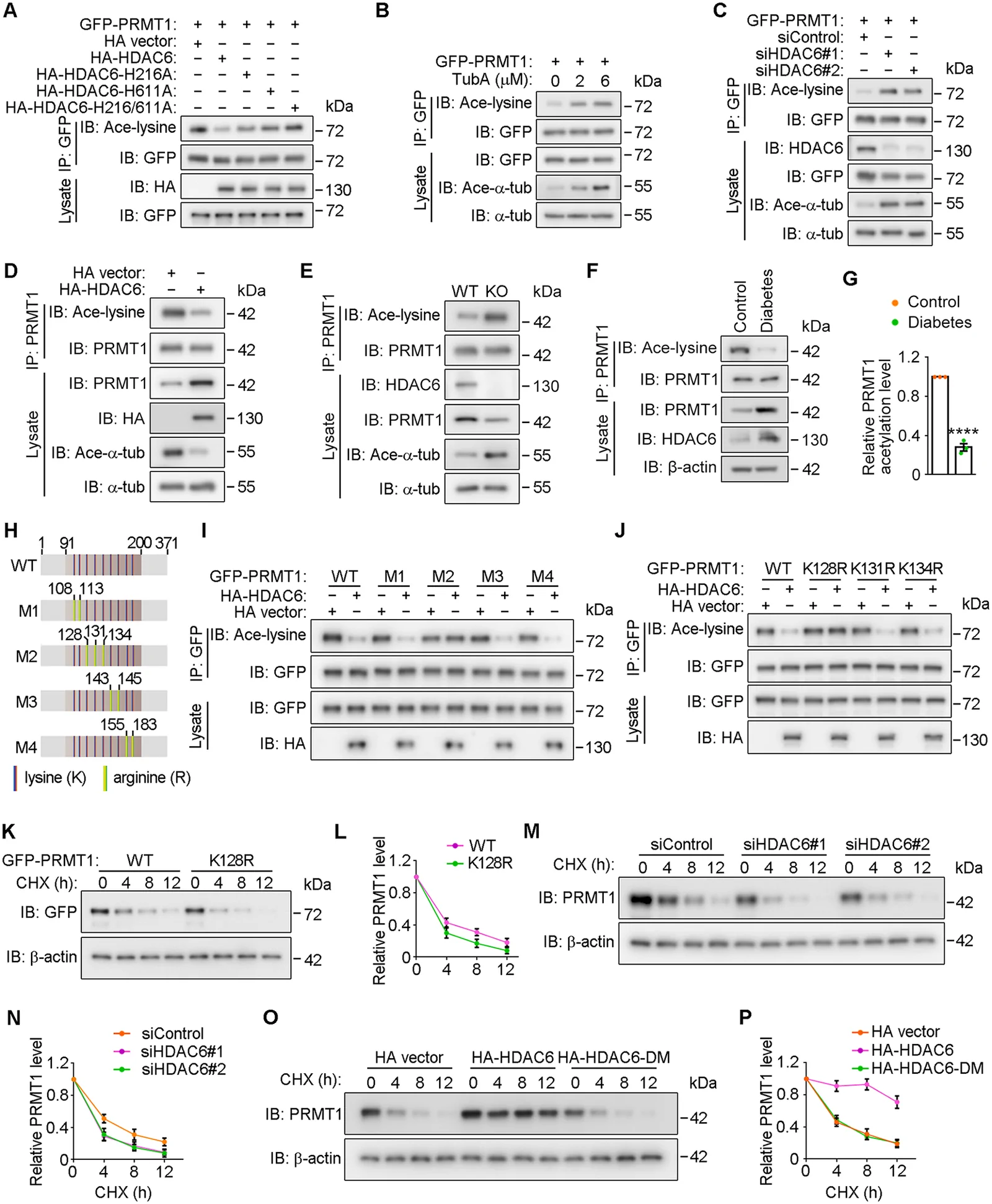
HDAC6-mediated deacetylation enhances PRMT1 stability. **(A)** Immunoprecipitation and immunoblotting to examine PRMT1 acetylation in cells transfected with HA vector, HA-HDAC6 or its inactive mutants, in the presence of GFP-PRMT1. **(B, C)** The acetylation of PRMT1 was analyzed by immunoprecipitation with GFP antibody followed by immunoblotting in cells transfected with GFP-PRMT1 and treated with or without tubastatin A (TubA, **B)**, and in cells transfected with control or HDAC6 siRNAs in presence of GFP-PRMT1 **(C)**. **(D)** Analysis of endogenous PRMT1 acetylation in cells transfected with HA vector or HA-HDAC6. **(E)** Analysis of endogenous PRMT1 acetylation in retinas from wild-type or *Hdac6* knockout mice. **(F, G)** Analysis of endogenous PRMT1 acetylation in retinas from control and diabetic mice, and the relative PRMT1 acetylation level was determined by densitometry. **(H)** Schematic diagram of PRMT1 wild-type and lysine-to-arginine (KR) mutants. **(I, J)** Identification of the site of PRMT1 deacetylation by HDAC6 by immunoprecipitation with the anti-GFP antibody followed by immunoblotting with the acetylated-lysine (Ace-lysine) antibody in cells transfected with GFP-PRMT1 wild-type or its KR mutants in the presence of HA-HDAC6 or HA vector. **(K, L)** Analysis of the stability of PRMT1 wild-type or its K128R mutant. **(M–P)** The protein stability of PRMT1 was examined in cells transfected with control or HDAC6 siRNAs **(M)**, and in cells transfected with HA vector, HA-HDAC6 wild-type or its inactive mutants **(O)**, and the relative PRMT1 level was determined by densitometry **(N, P)**. Data are presented as mean ± SEM. ****p < 0.0001. The data underlying this figure are available in S1 Data and S1 Raw Images.

We then sought to investigate the molecular mechanisms underlying the upregulation of PRMT1 under diabetic conditions. HDAC6, as a unique member of the HDAC family that primarily deacetylase several non-histone targets, is involved in various cellular activities, including cilium disassembly [31]. Notably, we observed that HDAC6 interacted and co-localized with PRMT1 at the centrosome. These promot us to hypothesize that PRMT1 might be a target of HDAC6-mediated deacetylation. To test this possibility, HEK293T cells were transfected with GFP-PRMT1 and HA-HDAC6 wild-type as well as its various mutants including H216A (histidine 216 mutated to alanine in the first deacetylase domain), H611A (histidine 611 mutated to alanine in the second deacetylase domain), and H216/611A (mutations of both histidine 216 and histidine 611 to alanines) [34,35] (Fig 5A). Our experiments revealed a significant reduction in PRMT1 acetylation in cells overexpressing wild-type HDAC6, and a slight reduction in cells transfected with H216A and H611A mutants (Fig 5A). However, overexpression of the H216/611A mutant did not affect PRMT1 acetylation (Fig 5A). Conversely, inhibition of HDAC6 deacetylase activity with tubastatin A, a specific inhibitor, or knockdown of HDAC6 with two different siRNAs, efficiently increased the acetylation of PRMT1 (Fig 5B and 5C). Consistent with these findings, the acetylation level of endogenous PRMT1 was significantly lower in HA-HDAC6 overexpressing cells but higher in retinas from *Hdac6* knockout mice compared to their controls (Fig 5D and 5E). Moreover, we found that PRMT1 protein levels were elevated in HDAC6-overexpressing cells but decreased in retinas from *Hdac6* knockout mice (Fig 5D and 5E), which aligns with the simultaneous upregulation of both proteins in the diabetic mice. Strikingly, in the retinas of diabetic mice, the acetylation of PRMT1 was markedly reduced compared to controls (Fig 5F and 5G), corresponding to the elevated protein level of HDAC6 in diabetic retinas. Together, these findings reveal that HDAC6 promotes the deacetylation and protein level of PRMT1 in the diabetic context.

To investigate the molecular mechanisms by which HDAC6 regulates PRMT1 protein levels, we sought to identify the specific site of deacetylation within PRMT1 by generating lysine-to-arginine (KR) mutations in various combinations within the 91–200 aa region of PRMT1 (Fig 5H). Immunoprecipitation experiments revealed that only the K128/131/134R mutations (replacement of lysine 128, 131, and 134 with arginines) impeded the deacetylation of PRMT1 by HDAC6 (Fig 5I), indicating that these three lysine residues serve as the sites for PRMT1 deacetylation. Further analysis of individual KR mutants demonstrated that the K128R mutation, but not the K131R or K134R mutation, completely abolished PRMT1 deacetylation by HDAC6 (Fig 5J), implying that K128 is the essential site for HDAC6-mediated deacetylation. Then, we explored whether HDAC6-mediated deacetylation affects PRMT1 stability. We found that the K128R mutant exhibited a shorter half-life than wild-type PRMT1 (Fig 5K and 5L). Furthermore, the half-life of PRMT1 was much shorter in HDAC6-depleted cells, and longer in cells overexpressing HDAC6 but not its catalytically inactive mutant, compared to the control groups (Fig 5M–5P). Collectively, these results suggest the existence of a positive feedback loop between PRMT1 and HDAC6 under diabetic conditions, where each enzyme protects the other from degradation, ensuring persistent cilium disassembly.

### Pharmacological disruption of the PRMT1-HDAC6 circuit restores ciliary homeostasis and ameliorates organ dysfunction

**Fig 6:**
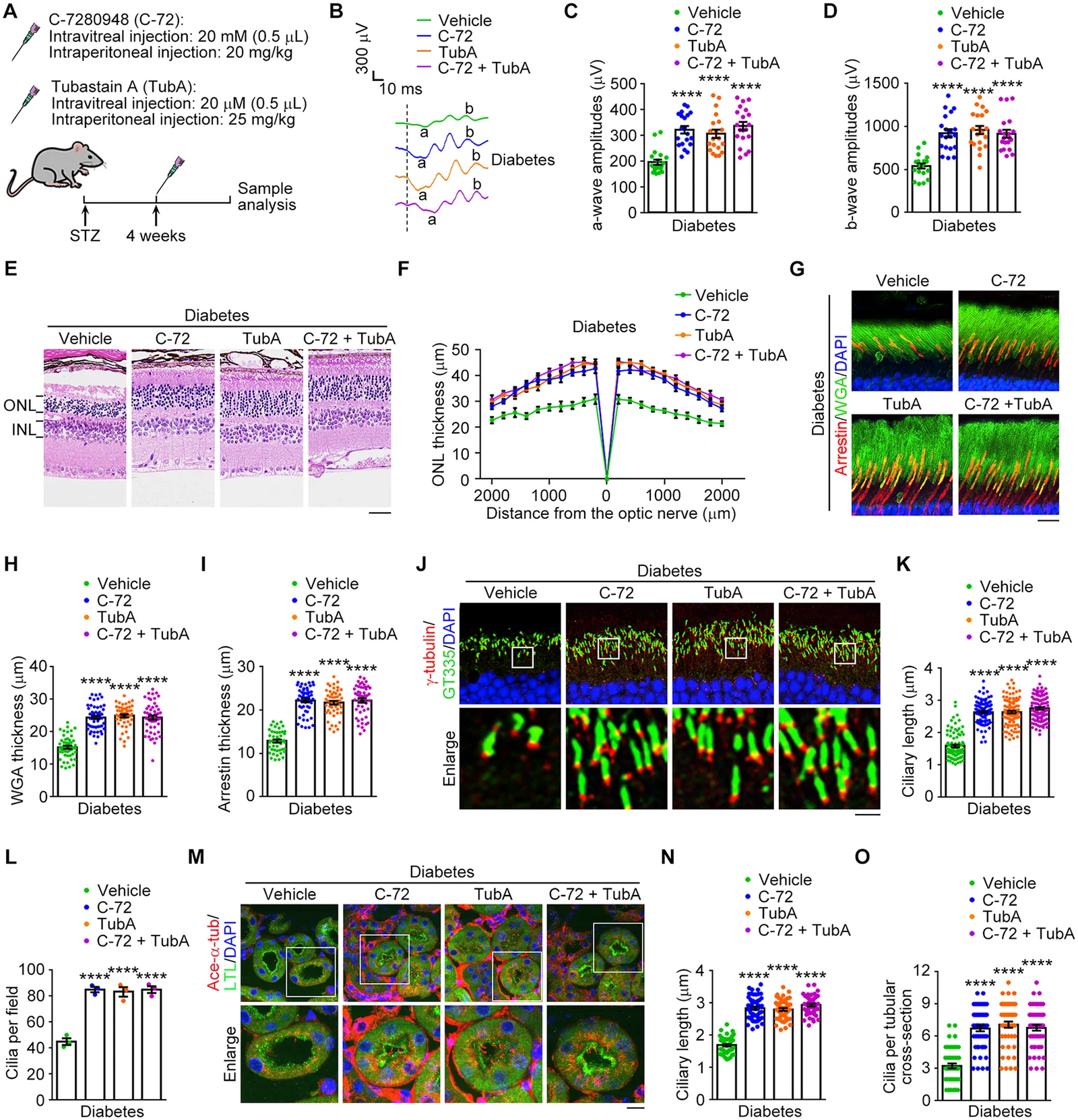
Inhibiting the PRMT1-HDAC6 feedback loop ameliorates pathological alterations in DR and DN. **(A)** Schematic illustration of intravitreal or intraperitoneal injection of C-7280948 and tubastatin A to inhibit PRMT1 an HDAC6 in diabetic mice, respectively. **(B–D)** ERG recordings (B) and measurement of retinal a-wave (C) and b-wave (D) amplitudes in diabetic mice following intravitreal injection with C-7280948, tubastatin A, or a combination of both inhibitors, vs. vehicle control (*n* = 20 mice from three independent experiments). **(E, F)** Photomicrographs (E) and quantification (F) of the retinal histology assessed by H&E staining in diabetic mice intravitreally injected with C-7280948, tubastatin A, or a combination of both inhibitors, vs. vehicle control (*n* = 3 independent experiments using a total of 24 mice). Scale bar, 30 μm. **(G–I)** Immunofluorescence images (G) and quantifications of the thickness of outer segment membranous discs of rods (H) and cones (I) from diabetic mice intravitreally injected with C-7280948, tubastatin A, or a combination of both inhibitors, vs. vehicle control (*n* = 50 fields from three independent experiments). Scale bar, 10 μm. **(J–L)** Immunofluorescence images (J) and quantifications of the length (K, *n* = 100 fields from three independent experiments) and density (L, *n* = 3 independent experiments) of ciliary axonemes in retinas from diabetic mice intravitreally injected with C-7280948, tubastatin A, or a combination of both inhibitors, vs. vehicle control. Scale bar, 2 μm. **(M–O)** Immunofluorescence images (M) and quantification of ciliary length (N) and density (O) in LTL^+^ proximal tubules from diabetic mice intraperitoneally injected with C-7280948, tubastatin A, or a combination of both inhibitors, vs. vehicle control (*n* = 50 renal tubules from three independent experiments). Scale bar, 6 μm. Data are presented as mean ± SEM. ****p < 0.0001. The data underlying this figure are available in S1 Data.

Given that our study has demonstrated the critical role of the positive feedback loop of PRMT1-HDAC6 in the pathogenesis of DR and DN, we aimed to investigate whether inhibiting this loop could alleviate the associated pathological changes. To achieve this, the diabetic mice were treated with C-7280948 and tubastatin A, to inhibit the activity of PRMT1 and HDAC6, respectively (Fig 6A). Our data showed a significant reduction in the levels of both HDAC6 and PRMT1 following the injection of C-7280948, tubastatin A, or both (S7A and S7B Fig). This result suggests that the hyperactive feedback loop between PRMT1 and HDAC6, which is closely associated with diabetic complications, was efficiently disrupted by PRMT1/HDAC6 inhibition.

To explore whether inhibition of this loop protects against retinal dysfunction related to DR, ERG analysis was performed for the diabetic mice. Notably, the decreased a-wave and b-wave amplitudes in diabetic mice were reversed by intravitreal injection of C-7280948, tubastatin A, or both (Fig 6B–6D). Correspondingly, our histopathological analysis of retinal morphology revealed a significant rescue of photoreceptor ONL thickness upon intravitreal injection of these inhibitors (Fig 6E and 6F). Additionally, inhibition of the PRMT1-HDAC6 loop effectively hindered the impairment of membranous discs and ciliary axonemes associated with DR (Fig 6G–6L). Thus, these findings suggest that inhibiting the reciprocal feedback loop of PRMT1 and HDAC6 protects mice from DR-associated retinal deficits.

We further examined the impact of inhibiting the PRMT1-HDAC6 loop on the pathological changes associated with DN. Consistent with their preventive effects in the retina, mice treated with C-7280948 and/or tubastatin A showed resistance to STZ-induced damages to renal glomerular and tubular structures, as evidenced by the decline in both the thickness of basement membrane and the luminal area of proximal tubules (S7C–S7F Fig). Additionally, the ciliary defects observed in proximal tubules under diabetic conditions were also improved by treatment with these inhibitors (Fig 6M–6O). Crucially, the elevated ACR observed in STZ-treated mice were also partially attenuated by the inhibitor treatments (S7G Fig). Taken together, these results demonstrate that the PRMT1-HDAC6 feedback loop is a potential therapeutic target for the systemic management of metabolic ciliopathies.

Since our findings indicate that ciliary disruption is a pivotal event in the progression of diabetic complications, we hypothesized that directly promoting ciliogenesis might ameliorate the pathological changes associated with DR and DN. To test this possibility, diabetic mice were treated with prostaglandin E2 (PGE2), a pharmacological agent known to promote ciliogenesis. We examined whether the ciliary defects induced by hyperglycemic stress could be relieved by this small-molecule compound. Indeed, PGE2 administration effectively restored ciliary length and incidence in both the retinal photoreceptors and renal tubules (S8A–S8F Fig). Next, we assessed whether promoting ciliogenesis could relieve the structural and functional deficits of the target tissues. Our results demonstrated that PGE2 treatment led to the preservation of retinal membranous disc and ONL thickness (S8G–S8K Fig), recovery of ERG a-wave and b-wave amplitudes (S8L–S8N Fig), and amelioration of renal glomerular and tubular abnormalities (S8O–S8R Fig). These results thus demonstrate that promoting ciliogenesis can ameliorate the tissue abnormalities induced by hyperglycemic stress, further supporting the potential of targeting ciliary homeostasis for the metabolic ciliopathies.

## Discussion

The prevalence of diabetic complications, particularly DR and DN, continues to rise globally. While microvasculopathy and glomerulosclerosis are established as the primary pathological features of these conditions, the underlying common cellular mechanisms that exacerbate multi-organ vulnerability remain incompletely understood. Our study identifies PRMT1-HDAC6-mediated ciliary disassembly as a novel and critical contributor that exacerbates retinal and renal vulnerability under hyperglycemic stress. This pathway may act as a pathological amplifier cooperating with traditional vascular and glomerular damage. We demonstrate that hyperglycemic stress triggers a self-reinforcing PTM circuit at the ciliary basal body, consisting of reciprocal PRMT1-mediated methylation and HDAC6-mediated deacetylation. This pathological loop refines the cell under a pro-disassembly state, driving the disassembly of the primary cilium, a sensory organelle essential for both phototransduction in the retina and mechanosensing in the kidney. Our findings provide a molecular mechanism that explains how chronic metabolic stress is translated into systemic organelle-level dysfunction. Additionally, inhibitors targeting this feedback loop could largely ameliorate pathological changes associated with DR and DN, suggesting that targeting the PRMT1-HDAC6 feedback loop could offer a promising therapeutic approach for managing these interconnected conditions.

PTMs are vital for increasing proteome diversity, enabling cells to respond promptly to internal and external cues, thereby maintaining tissue homeostasis [36–39]. Accumulating evidence demonstrates a critical role of various PTMs in the pathogenesis of ciliopathies [16,40]. For example, the UFMylation of kinesin family protein 11 (KIF11), a ciliary protein situated at the connecting cilium of photoreceptors, is crucial for maintaining photoreceptor cilium integrity and retinal homeostasis [41]. Similarly, protein polycystin 1, the product of the polycystic kidney disease gene *PKD1*, is modified by palmitoylation, contributing to its role in the pathogenesis of polycystic kidney disease [42]. In this work, we shed light on the disruption of cilia in the pathogenesis of DR and DN, and identify arginine methylation and deacetylation as key modifications that modulate ciliary homeostasis through the PRMT1-HDAC6 feedback loop. These findings enrich our understanding of the diverse regulatory mechanisms governing ciliary function and opens new avenues for investigating the role of PTMs in ciliopathies. By establishing a crosstalk between these two modifications and linking them to ciliary homeostasis, our work provides a foundation for developing therapeutic strategies targeting ciliary dysfunction in ciliopathies such as polycystic kidney disease and retinal degeneration.

Protein arginine methylation, catalyzed by PRMTs, is a common PTM in eukaryotes. This modification plays a role in various physiological processes by modulating the stability or localization of its targets [28]. Emerging evidence indicates that PRMTs play an essential role in various diseases. For instance, PRMT5-mediated neuronal death has been associated with Alzheimer’s disease [43], while PRMT6 is involved in polyglutamine diseases such as Huntington’s disease, spinobulbar muscular atrophy, dentatorubral-pallidoluysian atrophy, and spinocerebellar ataxias [44,45]. PRMT1 has been implicated in the pathogenesis of amyotrophic lateral sclerosis through its regulation of nuclear-cytoplasmic FUS localization [46]. These accumulating results, together with our finding that the upregulation of PRMT1 plays a crucial role in the pathological changes associated with diabetic complications, indicate that distinct PRMTs play different roles in diverse diseases. Moreover, our current results reveal that intravitreal injection of C-7280948, a selective inhibitor for PRMT1, largely ameliorates the pathological changes associated with diabetes, implying the potential value of pharmacologically targeting PRMT1 for diabetic prevention. Notably, clinical evidence further supports the relevance of PRMT1 in human diabetic complications. For example, the single-nucleotide polymorphism (rs3745468) in *PRMT1* gene has been associated with an increased incidence of proliferative DR in patients with type 2 diabetes mellitus [47], suggesting that genetic variations in *PRMT1* may influence individual susceptibility to microvascular damage under hyperglycemic stress.

HDAC6, a member of the HDAC family primarily located in the cytoplasm, plays a vital role in various cellular processes, including cilium disassembly [32,34,48]. Our previous research has demonstrated that HDAC6 facilitates cilium disassembly by deacetylating cortactin and α-tubulin [33]. Furthermore, the HDAC6-cilium axis is subject to regulation by a diverse range of proteins in different cellular contexts. For instance, the HEF1-Aurora A axis can activate HDAC6-dependent ciliary resorption by promoting its phosphorylation [49]. We have also shown previously that apoptosis signal-regulating kinase 1 (ASK1) and von Hippel–Lindau (VHL) mediate the crosstalk between HDAC6 phosphorylation and ubiquitination, affecting both the protein level and subcellular localization of HDAC6 at the photoreceptor cilium, thus contributing to the pathological retinal deficits associated with retinopathy of prematurity [50]. However, there is relatively limited information about the reciprocal feedback regulation of HDAC6. Our current findings reveal that HDAC6 undergoes methylation at R16 mediated by PRMT1, which increases HDAC6 stability and, in turn, facilitates PRMT1 stability by mediating its deacetylation at K128, creating a reinforcing cycle of regulation. This positive feedback loop between HDAC6 and PRMT1 provides novel insights into the regulatory network of these two proteins. Clinical evidence has demonstrated that HDAC6 is upregulated in diabetic nephropathy patients to drive podocyte injury [51]. Furthermore, human genomic data from large-scale metabolic GWAS have identified the *HDAC6* missense variant (rs61735967) as a significant metabolic quantitative trait locus for N6-acetyllysine levels [52], further linking the genetic variation of HDAC6 to human metabolic homeostasis.

Although our study has revealed the crosstalk of arginine methylation and deacetylation mediated by the PRMT1-HDAC6 feedback loop in the pathogenesis of DR and DN, several critical questions remain unresolved. For instance, while our study confirms that PRMT1 acts as a novel regulator of mammalian ciliary homeostasis, its downstream targets beyond HDAC6 remain an intriguing question. In *Chlamydomonas*, PRMT1 has been reported to interact with IFT molecules and show different localization in the flagella [30]. Whether mammalian PRMT1 modulates IFT proteins to drive ciliary resorption under metabolic stress requires further investigation. Notably, as HDAC6-mediated ciliary disassembly relies on the deacetylation of both α-tubulin and cortactin, its pathological function likely extends beyond the ciliary basal body to involve coordinated regulation across the cytoplasmic compartment. The PRMT1-HDAC6 complex enriched at the basal body may act as a critical scaffold that orchestrates broader cytoskeletal changes. Thus, exploring additional proteins whose functions are modulated under DR and DN conditions would be essential for elucidating the regulatory network of metabolic ciliopathies. Furthermore, it remains to be determined whether the PRMT1-HDAC6 feedback loop operates in other diabetic complications, such as diabetic cerebrovascular disease and diabetic neuropathy. Addressing these questions will significantly advance our understanding of the molecular mechanisms underlying the PRMT1-HDAC6 feedback loop in diabetic pathology. Additionally, assessing PRMT1 and HDAC6 protein levels in diabetic patients will provide critical insights into their pathophysiological roles and potential as therapeutic targets.

## Materials and methods

### Ethics statement

All animal experiments in this study were conducted in accordance with the recommendations in the Guide for the Care and Use of Laboratory Animals issued by the Ministry of Science and Technology of China, and were approved by the Animal Care and Use Committee of Shandong Normal University (permit number: AEECSDNU2025038). All animal care and use procedures adhered to the Regulations for the Administration of Affairs Concerning Experimental Animals approved by the Ministry of Science and Technology of China.

### Antibodies and reagents

Antibodies and reagents for immunofluorescence and immunohistochemistry staining include anti-γ-tubulin (T6557; Sigma-Aldrich), anti-acetylated α-tubulin (T6793; Sigma-Aldrich), anti-cone arrestin antibody (ab15282; Millipore), anti-polyglutamylated tubulin (GT335; AG20B-0020; AdipoGen Life Sciences), anti-PRMT1 (generated in our lab by immunizing rat with purified full length of PRMT1), anti-HDAC6 (AP1106a; Abcepta), anti-cone arrestin (ab15282; Millipore), anti-ARL13B (17711-1-AP; Proteintech), anti-LRP2 (GB112109; Servicebio), anti-THP (GB112167; Servicebio), biotinylated LTL (B-1325-2; Vector Laboratories), biotinylated DBA (B-1035-5; Vector Laboratories), Alexa Fluor 488-conjugated WGA (W11261; Thermo Fisher Scientific), Alexa Fluor 568 donkey anti-mouse IgG (H + L) (A10037; Thermo Fisher Scientific), Alexa Fluor 488 donkey anti-mouse IgG (H + L) (A21202; Thermo Fisher Scientific), Alexa Fluor 568 donkey anti-rabbit IgG (H + L) (A10042; Thermo Fisher Scientific), and Alexa Fluor 488 donkey anti-rabbit IgG (H + L) (A21206; Thermo Fisher Scientific), Alexa Fluor 568 donkey anti-rat IgG (H + L) (A78946; Thermo Fisher Scientific), DyLight 488-conjugated native streptavidin (ab134349; Abcam), and DAPI (D9542; Sigma-Aldrich). Antibodies and reagents for immunoprecipitation and immunoblotting include anti-HDAC6 (07-732; Millipore), anti-PRMT1 (2449S; Cell Signaling Technology), anti-β-actin (A5316; Sigma-Aldrich), anti-Flag (F3165; Sigma-Aldrich), anti-GST (ab9085; Abcam), anti-GFP (11814460001; Roche), anti-HA (H3663; Sigma-Aldrich); anti-ADMA (13522; Cell Signaling Technology), anti-acetylated-lysine (9441; Cell Signaling Technology), rabbit IgG (66467-1-Ig; Proteintech), goat anti-mouse IgG/HRP (SE131; Solarbio), and goat anti-rabbit IgG/HRP (SE134; Solarbio). Agarose beads for immunoprecipitation and GST pull-down including GFP nanoab agarose beads (GNA-25-500; NuoyiBio), protein A/G beads (20421; Thermo Fisher Scientific), anti-HA beads (A2095; Sigma-Aldrich), and glutathione resins (L00206; GenScript).

### Cell culture, plasmid transfection, and siRNAs

HEK293T, IMCD3 and RPE1 cells were obtained from the American Type Culture Collection (ATCC). HEK293T cells were cultured in Dulbecco’s modified Eagle medium (DMEM), while IMCD3 and RPE1 cells were maintained in DMEM: F12 (1:1) medium, supplemented with 10% fetal bovine serum (FBS, Biological Industries, Bioind). Plasmids encoding HDAC6 wild-type and mutants were described previously [53]. The full-length and fragment-truncated mutant PRMT1 cDNAs were cloned into the pEGFP-C1 vector. Mutations converting lysine to arginine in PRMT1, or arginine to lysine in HDAC6 were generated using PCR and site-directed mutagenesis. The sequences of specific siRNAs targeting PRMT1, HDAC6, or non-targeting controls used in this study were as follows: siHDAC6#1 (5′- GCAGUUAAAUGAAUUCCAU-3′), siHDAC6#2 (5′- GGAGUUAACUGGCAGGCAU-3′; siPRMT1#1, 5′-GCCUACUUCAACAUCGAGU-3′), siPRMT1#2 (5′- CGGCAGUACAAAGACUACA-3′), and the control siRNA (5′-CGUACGCGGAAUACUUCGA-3′). For plasmid transfections, HEK293T and IMCD3 cells were seeded in antibiotic-free plates, and the transfection mixture containing plasmids and polyethyleneimine (PEI, 23966-1; Polysciences) or Lipofectamine 3000 (L3000-015; Invitrogen) was added to the culture medium after cell adherence. The medium was replaced with fresh medium supplemented with 10% FBS 12 hours post-transfection. siRNA transfections were performed using Lipofectamine RNAiMAX (13778030; Invitrogen), following the manufacturer’s instructions.

### Mouse strains

The *Prmt1* conditional knockout (*Prmt1*^*flox/flox*^*; CAG-Cre-ERT2*) and *Hdac6* knockout mice in the C57BL/6 background were produced and genotyped as described previously [50,54]. Specifically, conditional *Prmt1* knockout mice were generated using a floxed allele system (*Prmt1*^*flox/flox*^). To achieve systemic deletion, *Prmt1*^*flox/flox*^ mice were crossed with the *CAG-Cre-ERT2* transgenic line. To induce PRMT1 depletion, *Prmt1*^*flox/flox*^*; CAG-Cre-ERT2* mice were injected intraperitoneally with tamoxifen (T5648; Sigma) at a dose of 70 mg/kg body weight for five consecutive days. *Prmt1*^*flox/flox*^ mice (lacking the *CAG-Cre-ERT2* allele) receiving the identical tamoxifen treatment served as the wild-type controls for the study. Both male and female mice were used and sex balanced for all experiments.

### Diabetic mouse model

The diabetic mouse model was established following our previously described protocol [55]. Briefly, 8- to 10-week-old male C57BL/6 mice were fasted for four hours prior to the administration of STZ (572201; Millipore), which was dissolved in 0.1 M citrate buffer (pH 4.5). For the *Prmt1* KO mice, the STZ induction protocol was initiated exactly 7 days after the final tamoxifen injection, a time point confirmed to ensure the complete depletion of PRMT1 protein prior to the induction of hyperglycemic stress. The mice received daily intraperitoneal injections of STZ (55 mg/kg) or an equivalent volume of citrate buffer (as a control) for five consecutive days. After 48 hours, diabetes was confirmed in the STZ-treated mice by determination of blood sugar of more than 250 mg/dL; otherwise, the mice were excluded. The diabetic mice were randomly divided into five groups: STZ + vehicle, STZ + C-7280948, STZ + tubastatin A, STZ + C-7280948 + tubastatin A, and STZ + PGE2. STZ-treated mice received weekly intravitreal or intraperitoneal injections of C-7280948 (HY-15890; MedChemExpress), tubastatin A (sml0044; Sigma-Aldrich), PGE2 (P0409; Sigma-Aldrich) or an equivalent volume of vehicle, starting from four weeks post-STZ treatment. For intravitreal injections, mice were anesthetized using 2% isoflurane inhalation, followed by the application of 0.5% oxybuprocaine hydrochloride (Santen Pharmaceuticals) and 0.5% tropicamide phenylephrine (Santen Pharmaceuticals) to the corneal surface for local anesthesia and pupil dilation. Mice were intravitreally injected with 0.5 μL of solution (C-7280948, 20 mM; tubastatin A, 20 μM; PGE2, 50 μM) or intraperitoneally injected with these inhibitors (C-7280948, 20 mg/kg; tubastatin A, 25 mg/kg; PGE2, 4 mg/kg).

### ERG analysis

Mice received 1% pentobarbital sodium by intraperitoneal injection to induce anesthesia. The corneas of the mice were desensitized using 0.5% oxybuprocaine hydrochloride, and their pupils were dilated using 0.5% tropicamide phenylephrine. A feedback temperature controller (TC-100, Eaton) was used to maintain the body temperature of the mice at a constant level of 37 ± 0.5 °C. Subcutaneous reference electrodes from the RetiMINER-C visual electrophysiological system (IRC Technologies, Bangkok, Thailand) were positioned below the ears. A gold wire electrode was placed on the tail, while the recording electrodes were placed on the corneal surface of each eye. Scotopic ERG responses were recorded in response to white flash stimuli (3 cd·s/m^2^). The a-wave amplitude was measured as the difference between the baseline and the lowest point of the a-wave, while the b-wave amplitude was measured from the lowest point of the a-wave to the highest point of the b-wave.

### Histopathological analysis

Samples were fixed by immersing them in 4% paraformaldehyde solution at 4 °C overnight. Following this, the tissues were further fixed for additional 2 hours at room temperature. The fixed samples were then embedded in paraffin, and 4-μm sections of the samples were prepared. To ensure consistency, sections from the same position within the retina and kidney that were embedded in the same direction were chosen for analysis. Using a DM3000 microscope (Leica, Wezlar, Germany), the sections were examined and photographs were captured to document the findings.

### Immunofluorescence staining

Mice were humanely euthanized using an overdose of pentobarbital sodium. To prepare the samples for membranous discs staining, a fixative solution consisting of 4% paraformaldehyde, 80 mM PIPES (pH 6.8), 5 mM EGTA, and 2 mM MgCl_2_ was perfused through the heart. The eyes were swiftly removed and post-fixed overnight in the same fixative solution at 4 °C. After removing the cornea and lens, the eye cups were embedded in 5% low-melt agarose and sectioned into 80 μm slices using a vibratome (Lecia). Retinal sections were then treated with a blocking solution containing 7% goat serum and 0.5% triton X-100 for 1 hour at room temperature. Subsequently, the sections were incubated with Alexa Fluor 488-conjugated WGA, washed in PBS, and exposed to anti-cone arrestin antibody overnight at 4 °C. This was followed by staining with Alexa Fluor 568-conjugated secondary antibody at room temperature for 1 hour. After washing, the sections were stained with DAPI and mounted onto slides in glycerol. Images were captured using a Dragonfly 200 confocal imaging system (Andor Technology, Belfast, UK).

For staining of cilia, the retina or kidney samples were fixed with 4% paraformaldehyde for a short duration (approximately 30 seconds) at room temperature. Cryo-embedding in Tissue-Tek OCT was performed, and 10-μm frozen sections were prepared using a freezing microtome (CM3050S; Lecia). These sections were then incubated with antibodies against polyglutamylated tubulin, acetylated tubulin and γ-tubulin overnight at 4 °C, followed by washing. Slides were incubated with Alexa Fluor 568-conjugated or Alexa Fluor 488-conjugated secondary antibodies for 1 hour at room temperature. After washing, slides were stained with DAPI and imaged using a Leica SP8 confocal microscope. Analysis of the images was performed using the 3D-analysis tool in Leica Application Suite X software.

Lectin staining was performed according to the manufacturer’s instructions. Briefly, renal tissue sections were fixed in acetone and subsequently blocked with 4% BSA. Endogenous biotin was quenched using the Streptavidin/Biotin Blocking Kit (SP-2002, Vector Laboratories) following the manufacturer’s protocol. Sections were then incubated with biotinylated LTL or biotinylated DBA, followed by incubation with DyLight 488-conjugated native streptavidin.

### Transmission electron microscopy

To perform transmission electron microscopy, the eyeballs were fixed using a solution containing 0.25% glutaraldehyde in 0.1 M sodium cacodylate for 4 hours at room temperature. Following removal of the lens and cornea, the eye cups were further fixed in the same solution but at 4 °C overnight. Subsequently, post-fixation was carried out using 1% osmium tetroxide for 1 hour at room temperature. The retinal samples were then dehydrated using a series of ethanol concentrations, infiltrated with resin, embedded in Spurr low viscosity resin, and cured for three days at 65 °C. Ultrathin sections measuring 50 nm were prepared and stained with uranyl acetate and lead citrate, as established in previous studies. Finally, images were captured using an HT-7800 transmission electron microscope (Hitachi, Tokyo, Japan) operated at 80 kV.

To accurately define and quantify structural abnormalities in photoreceptors, we assessed their ultrastructure based on three primary criteria: (1) axonemal defects, identified as the shortening or absence of the ciliary axoneme; (2) membranous disc disorganization, characterized by the loss of regular parallel stacking, structural bending, or fragmentation; and (3) dimensional and spacing irregularities, defined as abnormally widened inter-disc spacing, shortened individual discs, or altered overall thickness of the outer segment. A photoreceptor cell was categorized as structurally abnormal if it exhibited at least one of these morphological defects. For statistical quantification, the percentage of structural abnormalities was determined by calculating the ratio of abnormal photoreceptor cells to the total number of cells analyzed in each independent experiment.

### Mass spectrometry

Quantitative proteomic analysis of mouse retinas was conducted by PTM Biolabs (Hangzhou, China). Briefly, retina samples obtained from both STZ-treated and control mice were rapidly frozen using liquid nitrogen and subsequently ground into fine powder. The powder was then lysed in a lysis buffer consisting of 8 M urea and 1% protease inhibitor cocktail, followed by sonication on ice using a high-intensity ultrasonic processor (Scientz). The resulting mixture underwent centrifugation at 4 °C, 12,000*g* for 10 min to eliminate cellular debris. Protein concentration was determined using a BCA assay kit. Subsequently, the proteins were digested with trypsin (Promega) to generate peptides, which were subsequently desalted, vacuum-dried, and processed for TMT labeling as per the manufacturer’s instructions of the TMT kit (Thermo Fisher Scientific). The tryptic peptides were subsequently fractionated using high pH reverse-phase high-pressure liquid chromatography (HPLC) with a Thermo Betasil C18 column. This facilitated downstream analysis using liquid chromatography coupled with tandem mass spectrometry (LC–MS/MS).

### Immunoprecipitation and immunoblotting

For protein extraction, mouse retinal tissues or HEK293T cells were lysed using a lysis buffer containing Tris (50 × 10^−3^ M, pH 7.5), NaCl (150 × 10^−3^ M), EDTA (1 × 10^−3^ M), glycerin (3%), NP40 (1%), and a protease inhibitor cocktail (Roche, Basel, Switzerland). The samples were then sonicated and centrifuged at 15,000 rpm for 20 min at 4 °C. Immunoprecipitation was conducted by incubating the lysates with agarose beads coated with primary antibodies for 4 hours at room temperature or overnight at 4 °C. The beads were washed five times, and the proteins bound to the beads were analyzed using SDS-PAGE and immunoblotting. For immunoblotting, the proteins were separated by SDS-PAGE and transferred onto polyvinylidene difluoride membranes (Millipore). These membranes were then blocked using a solution of Tris-buffered saline containing 0.2% Tween-20 and 5% non-fat milk. Subsequently, the membranes were incubated with primary antibodies, diluted in the blocking buffer, for 2 hours at room temperature or overnight at 4 °C. After five washes in Tris-buffered saline with Tween-20, the membranes were incubated with secondary antibodies conjugated to horseradish peroxidase for 45 min at room temperature. The bound antibodies were detected using an enhanced chemiluminescence detection reagent. Each experiment was repeated at least three times, and the intensity of the immunoblot bands was quantified using the ImageJ software.

### In vitro methylation assay

In vitro methylation assay was performed as follows. Briefly, 0.5 μg purified GST-HDAC6 protein (ab42632; Abcam) were incubated with various concentrations of recombinant PRMT1 (0.5, 1, or 2 μg, respectively, 31,411; Proteintech) in 30 μL methylation reaction buffer (20 mM Tris-HCl (pH 8.0), 200 mM NaCl, 0.4 mM EDTA and 1 mM S-adenosyl-L-methylmethionine) for 90 min at 30 °C. The methylation of HDAC6 was measured following GST pull-down and immunoblotting.

### RT-qPCR analysis

Retinal samples or cells were used for the isolation of total RNAs following the manufacturer’s protocol using the TRIzol reagent (Invitrogen). The isolated RNA was then subjected to cDNA synthesis using M-MLV reverse transcriptase (Promega, Madison, WI). For quantitative real-time PCR analysis, triplicate reactions were performed using the Power SYBR Green PCR Master Mix Kit (Applied Biosystems, Waltham, MA) and a 7500 HT Sequence Detection System. To ensure accurate normalization, GAPDH was used as a control gene, and the mRNA level of HDAC6 was normalized to that of GAPDH. The primers used for amplification were as follows: human HDAC6 (5′-GAGGGAGAACTCCGTGTCCTA-3′ and 5′-AATGCCATCCATAAGACTGTGC-3′), human GAPDH (5′-ATGAGGTCCACCACCCTGTT-3′ and 5′-ATCACTGCCACCCAGAAGAC-3′), mouse PRMT1 (5′-ACTGCCTCTTCTACGAGTCCA-3′ and 5′-TGCACGTAGTCATTCCGCTT-3′), mouse HDAC6 (5′-GCGCACACTTCTATCC-3′ and 5′-TCCAGCAATGACTTGGGCAT-3′), mouse GAPDH (5′-AGGTCGGTGTGAACGGATTTG-3′ and 5′-GGGGTCGTTGATGGCAACA-3′).

### Statistical analysis

Statistical analysis was performed using GraphPad Prism 8.0 software (GraphPad Software, La Jolla, CA). To ensure the validity of statistical conclusions, different animals were used for independent experiments to avoid pseudo-replication. Each experiment was performed on distinct biological replicates, and data points were derived from separate samples. Experimental data are expressed as mean ± SEM. Differences between two groups were assessed using Student *t* test for normally distributed data, while comparisons among three or more groups were made using two-way analysis of variance (ANOVA). Non-parametric tests were utilized for data that did not follow a normal distribution. Statistical significance was set at a *p*-value less than 0.05.

## Supporting information

S1 Fig Hyperglycemic stress induces ultrastructural ciliary abnormalities and ciliary disassembly. **(A)** Quantifications of the INL thickness assessed by H&E staining in diabetic and control mice. **(B, C)** Photomicrographs (B) and quantification of the inner luminal area of distal tubules (C) assessed by immunohistochemical staining for Tamm-Horsfall urinary glycoprotein (THP) from diabetic and control mice. (*n* = 30 fields from three independent experiments). Scale bar, 10 μm. **(D)** Transmission electron microscopy images of cross sections of photoreceptors in diabetic and control mice. Scale bars, 0.1 μm (upper) and 0.5 μm (bottom). **(E)** Quantification of the percentage of abnormal structures in photoreceptor ciliary axoneme and membranous discs from diabetic and control mice (*n* = 3 independent experiments). **(F–H)** Immunofluorescence images (F) and quantifications of ciliary length (G) and density (H) in kidney stained with antibody against acetylated α-tubulin, biotinylated Dolichos biflorus agglutinin (DBA), and DAPI from diabetic and control mice (*n* = 50 renal tubules from three independent experiments). Scale bar, 10 μm. **(I–K)** Immunofluorescence images (I) and quantifications of ciliary length (J, *n* = 50 fields from three independent experiments) and ciliary density (K, *n* = 3 independent experiments) in RPE1 and IMCD3 cells. Cells were treated with or without glucose (50 mM) for 24 hours under serum starvation conditions, and stained with antibodies against ARL13B and α-tubulin. Scale bar, 2 μm. Data are presented as mean ± SEM. **p < 0.01, ***p < 0.001, ****p < 0.0001; ns, not significant. The data underlying this figure are available in S1 Data.
(TIF)

S2 Fig Depletion of PRMT1 or HDAC6 ameliorates pathological changes associated with DR and DN. **(A)** Immunoblot analysis of HDAC6, PRMT1, and β-actin in IMCD3 cells treated with or without glucose (50 mM) for 24 hours under serum starvation conditions. **(B, C)** Quantitative RT-PCR analysis of relative *Prmt1* and *Hdac6* mRNA levels in retinas and kidneys from control or diabetic mice (*n* = 3 independent experiments). **(D–G)** Immunoblot analysis of HDAC6, PRMT1, and β-actin in retinas and kidneys from *Hdac6* knockout (D, E) or *Prmt1* conditional knockout (F, G) mice and their respective controls. **(H, I)** Quantifications of the ONL thickness assessed by H&E staining in *Hdac6* knockout or *Prmt1* conditional knockout mice and their respective controls under diabetic conditions. **(J, K)** Photomicrographs (J) and quantifications of basement membrane thickness (K) from the renal histology assessed by H&E staining in *Hdac6* knockout or *Prmt1* conditional knockout mice and their respective controls under diabetic conditions (*n* = 30 fields from three independent experiments). Scale bar, 20 μm. **(L, M)** Photomicrographs (L) and quantifications of the inner luminal area of proximal tubules (M) assessed by immunohistochemical staining for LRP2 in *Hdac6* knockout or *Prmt1* conditional knockout mice and their respective controls under diabetic conditions (*n* = 30 fields from three independent experiments). Scale bars, 10 μm. **(N)** Quantification of ACR in *Hdac6* knockout or *Prmt1* conditional knockout mice and their respective controls under control or diabetic conditions (*n* = 12 mice from three independent experiments). Data are presented as mean ± SEM. ***p < 0.001, ****p < 0.0001; ns, not significant. The data underlying this figure are available in S1 Data and S1 Raw Images.
(TIF)

S3 Fig Normal glycemic control and retinal homeostasis are preserved in *Prmt1* and *Hdac6* knockout mice. **(A)** Quantification of blood glucose levels in *Hdac6* knockout or *Prmt1* conditional knockout mice and their respective controls under control or diabetic conditions (*n* = 12 mice from three independent experiments). **(B–D)** Immunofluorescence images (B) and quantifications of ciliary axoneme length (C, *n* = 100 fields from three independent experiments) and ciliary density (D, *n* = 3 independent experiments) in retinas stained with antibodies against GT335 and α-tubulin from *Hdac6* knockout or *Prmt1* conditional knockout mice and their respective controls. Scale bar, 2 μm. **(E–G)** Immunofluorescence images (E) and quantifications of membranous disc thickness in rods stained with WGA (F) and cones stained with the anti-cone arrestin antibody (G) from *Hdac6* knockout or *Prmt1* conditional knockout mice and their respective controls (*n* = 50 fields from three independent experiments). Scale bar, 10 μm. **(H–J)** Photomicrographs (H) and quantifications (I, J) of the retinal histology assessed by H&E staining in *Hdac6* knockout or *Prmt1* conditional knockout mice and their respective controls. Scale bar, 40 μm. **(K–M)** ERG recordings (K) and measurements of retinal a-wave (L) and b-wave (M) amplitudes were performed for *Hdac6* knockout or *Prmt1* conditional knockout mice and their respective controls (*n* = 24 eyes from three independent experiments). Data are presented as mean ± SEM. ns, not significant. The data underlying this figure are available in S1 Data.
(TIF)

S4 Fig Depletion of PRMT1 or HDAC6 does not alter renal structural and functional integrity. **(A–C)** Immunofluorescence images (A) and quantifications of ciliary length (B) and density (C) in kidney stained with antibody against acetylated α-tubulin, biotinylated LTL, and DAPI from *Hdac6* knockout or *Prmt1* conditional knockout mice and their respective controls (*n* = 50 renal tubules from three independent experiments). Scale bar, 2 μm. **(D, E)** Photomicrographs (D) and quantification of basement membrane thickness (E) from the renal histology assessed by H&E staining from *Hdac6* knockout or *Prmt1* conditional knockout mice and their respective controls (*n* = 30 fields from three independent experiments). Scale bars, 20 μm. **(F, G)** Photomicrographs (F) and quantification of the inner luminal area of proximal tubules (G) assessed by immunohistochemical staining for LRP2 in *Hdac6* knockout or *Prmt1* conditional knockout mice and their respective controls (*n* = 30 fields from three independent experiments). Scale bars, 10 μm. Data are presented as mean ± SEM. **p < 0.01; ns, not significant. The data underlying this figure are available in S1 Data.
(TIF)

S5 Fig PRMT1 interacts with HDAC6. **(A)** Immunofluorescence staining of retinal photoreceptors in wild-type and *Prmt1* conditional knockout mice with antibodies against α-tubulin and PRMT1. Scale bar, 2 μm. **(B, C)** Quantifications of relative PRMT1 and HDAC6 centrosomal localization in photoreceptors from control and diabetic mice (*n* = 3 independent experiments). **(D, E)** Examination of the interaction of PRMT1 and HDAC6 in retinal (D) and renal (E) lysates by immunoprecipitation with the HDAC6 antibody followed by immunoblotting. **(F, G)** Immunoprecipitation and immunoblotting showing the interaction of PRMT1 with HDAC6 in IMCD3 cells. **(H)** Immunoprecipitation and immunoblotting showing the interaction of GFP-HDAC6 with Flag-PRMT1 in HEK293T cells. Data are presented as mean ± SEM. ns, not significant. The data underlying this figure are available in S1 Data and S1 Raw Images.
(TIF)

S6 Fig PRMT1-mediated arginine methylation enhances HDAC6 stability and promotes ciliary disassembly. **(A)** Immunoprecipitation and immunoblotting analysis of the asymmetric dimethylation of endogenous HDAC6 in mouse renal tissues. **(B, C)** Analysis of the asymmetric dimethylation of endogenous HDAC6 in renal tissues from wild-type and *Prmt1* conditional knockout mice (B), and from control and diabetic mice (C). **(D, E)** Immunoblot analysis of HDAC6 and β-actin in cells transfected with GFP vector, GFP-PRMT1 or its catalytically inactive mutant, and the relative HDAC6 level were determined by densitometry (*n* = 3 independent experiments). **(F)** Quantitative RT-PCR analysis of relative *Hdac6* mRNA level in cells transfected with GFP vector, GFP-PRMT1 or its catalytically inactive mutant (*n* = 3 independent experiments). **(G)** HDAC6 protein stability was examined in cells transfected with GFP vector, GFP-PRMT1 or its catalytically inactive mutant. **(H–J)** Immunofluorescence images (H) and quantifications of ciliary length (I, *n* = 30 cells from three independent experiments) and ciliary density (J, *n* = 3 independent experiments) in IMCD3 cells. Cells were transfected with GFP vector, GFP-HDAC6 wild-type, or the R16K mutant, followed by serum starvation for 24 hours, and stained with antibodies against acetylated α-tubulin and DAPI. Scale bar, 2 μm. **(K–M)** Immunofluorescence images (K) and quantifications of ciliary length (L, *n* = 30 cells from three independent experiments) and ciliary density (M, *n* = 3 independent experiments) in IMCD3 cells. Cells were cultured in serum-free medium for 24 hours to induce ciliogenesis, followed by culturing in serum-fed medium with or without C-7280948 to induce ciliary disassembly. Scale bar, 3 μm. Data are presented as mean ± SEM. *p < 0.05, ***p < 0.001, ****p < 0.0001; ns, not significant. The data underlying this figure are available in S1 Data and S1 Raw Images.
(TIF)

S7 Fig Inhibition of the PRMT1-HDAC6 feedback loop alleviates renal deficits related to DN. **(A, B)** Immunoblot analysis of HDAC6, PRMT1, and β-actin in retinas and kidneys from diabetic mice treated with C-7280948, tubastain A, or a combination of both inhibitors, versus vehicle control. **(C, D)** Photomicrographs (C) and quantification of basement membrane thickness (D) from the renal histology assessed by H&E staining in diabetic mice intraperitoneally injected with C-7280948, tubastain A, or a combination of both inhibitors, versus vehicle control (*n* = 50 fields from three independent experiments). Scale bar, 20 μm. **(E, F)** Photomicrographs (E) and quantification of the inner luminal area of proximal tubules (F) assessed by immunohistochemical staining for LRP2 in diabetic mice intraperitoneally injected with C-7280948, tubastain A, or a combination of both inhibitors, versus vehicle control (*n* = 30 fields from three independent experiments). Scale bars, 8 μm. **(G)** Quantification of ACR in diabetic mice intraperitoneally injected with C-7280948, tubastatin A, or a combination of both inhibitors, versus vehicle control (*n* = 12 mice from three independent experiments). Data are presented as mean ± SEM. ****p < 0.0001. The data underlying this figure are available in S1 Data and S1 Raw Images.
(TIF)

S8 Fig Promoting ciliogenesis ameliorates the pathological changes related to DR and DN. **(A–C)** Immunofluorescence images (A) and quantifications of the length (B, *n* = 100 fields from three independent experiments) and density (C, *n* = 3 independent experiments) of ciliary axonemes in retinas from diabetic mice intravitreally injected with prostaglandin E2 (PGE2) versus vehicle control. Scale bar, 2 μm. **(D–F)** Immunofluorescence images (D) and quantifications of ciliary length (E) and density (F) in LTL^+^ proximal tubules from diabetic mice intraperitoneally injected with PGE2 versus vehicle control (*n* = 50 renal tubules from three independent experiments). Scale bar, 2 μm. **(G–I)** Immunofluorescence images (G) and quantifications of the thickness of outer segment membranous discs of rods (H) and cones (I) from diabetic mice intravitreally injected with PGE2 versus vehicle control (*n* = 50 fields from three independent experiments). Scale bar, 10 μm. **(J, K)** Photomicrographs (J) and quantification (K) of the retinal histology assessed by H&E staining in diabetic mice intravitreally injected with PGE2 versus vehicle control (*n* = 3 independent experiments). Scale bars, 30 μm. **(L–N)** ERG recordings (L) and measurement of retinal a-wave (M) and b-wave (N) amplitudes in diabetic mice following intravitreal injection with PGE2 versus vehicle control (*n* = 24 mice from three independent experiments). **(O, P)** Photomicrographs (O) and quantification of basement membrane thickness (P) from the renal histology assessed by H&E staining in diabetic mice intraperitoneally injected with PGE2 versus vehicle control (*n* = 50 fields of three independent experiments). Scale bar, 20 μm. **(Q, R)** Photomicrographs (Q) and quantification of the inner luminal area of proximal tubules (R) assessed by immunohistochemical staining for LRP2 in diabetic mice intraperitoneally injected with PGE2 versus vehicle control (*n* = 30 fields from three independent experiments). Scale bars, 10 μm. Data are presented as mean ± SEM. *p < 0.05, ****p < 0.0001. The data underlying this figure are available in S1 Data.
(TIF)

S1 Table List of differentially regulated proteins identified in retinas and kidneys from control and diabetic mice. (XLSX)

S1 Data Numerical data for all figures in this study. (XLSX)

S1 Raw Images Raw images for all blots in this study. (PDF)

## Abbreviations

ACR: albumin-to-creatinine ratio
ADMA: asymmetric dimethylarginine
ANOVA: analysis of variance
ARL13B: ADP-ribosylation factor-like 13 B
ASK1: apoptosis signal-regulating kinase 1
ATCC: American Type Culture Collection
DMEM: Dulbecco’s modified Eagle medium
DN: diabetic nephropathy
DR: diabetic retinopathy
ERG: electroretinography
FBS: fetal bovine serum
HDAC6: histone deacetylase 6
HPLC: high-pressure liquid chromatography
H&E: hematoxylin and eosin
IFT88: intraflagellar transport 88
LC–MS/MS: liquid chromatography coupled with tandem mass spectrometry
LRP2: lipoprotein receptor-related protein 2
ONL: outer nuclear layer
PGE2: prostaglandin E2
PRMT1: protein arginine methyltransferase 1
PTMs: post-translational modifications
RT-qPCR: quantitative reverse transcription polymerase chain reaction
STZ: streptozotocin
THP: Tamm–Horsfall protein
VHL: von Hippel–Lindau
WGA: wheat germ agglutinin
