# Supplementary material for: Hyperglycemic stress aggravates diabetic retinopathy and nephropathy by promoting cilium disassembly via a deacetylation- and methylation-mediated regulatory mechanism
Source: PLoS Biol. 2026 Sep 3;24(9):e3003975. doi: 10.1371/journal.pbio.3003975 (PMC13541193; doi:10.1371/journal.pbio.3003975)

Fig. 2C

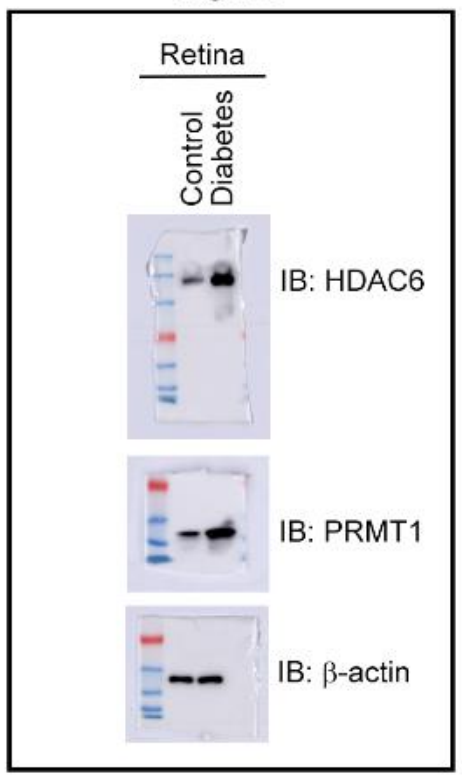

Fig. 2D

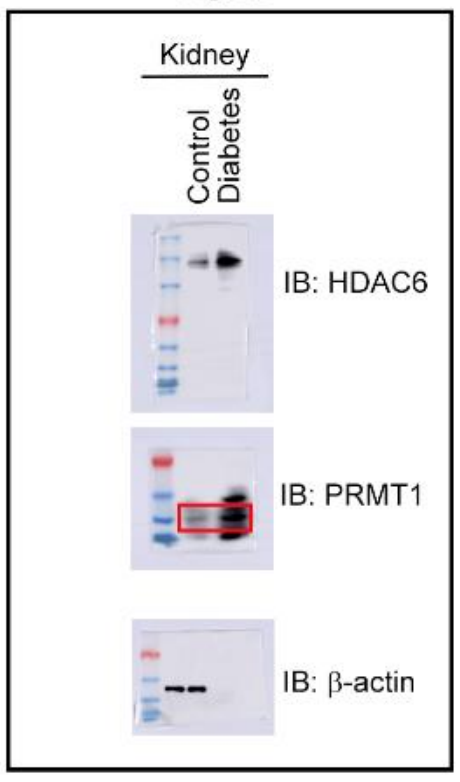

Fig. 3E

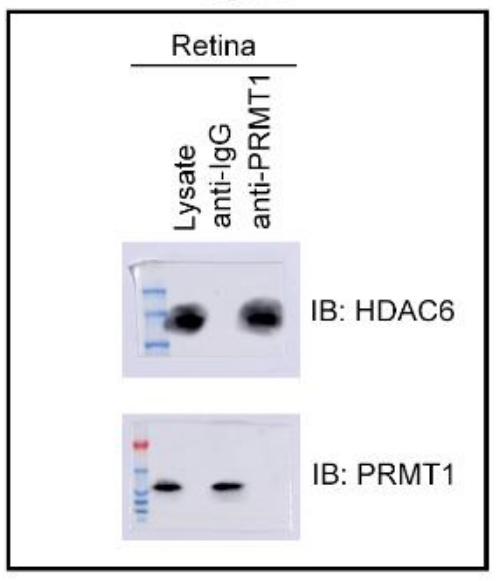

Fig. 3F

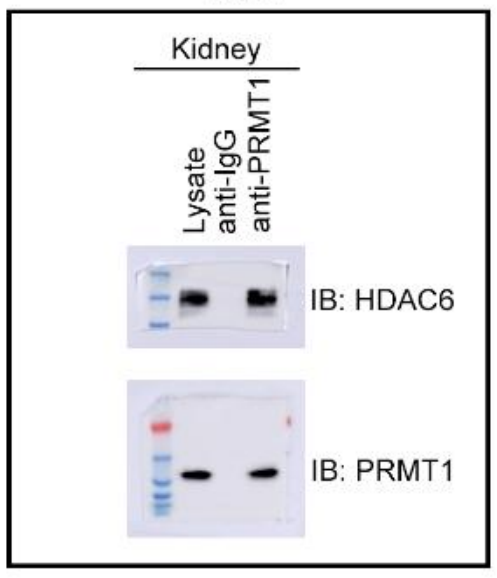

Fig. 3G

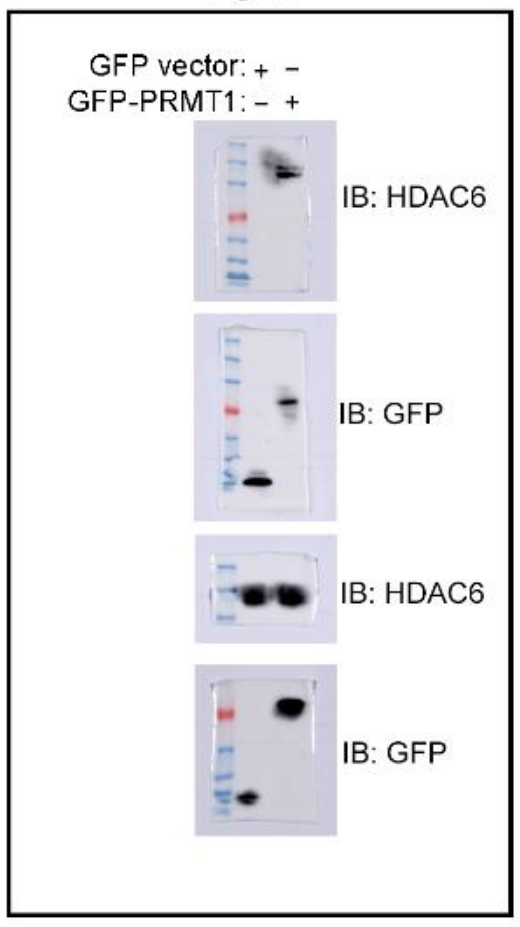

Fig. 3H

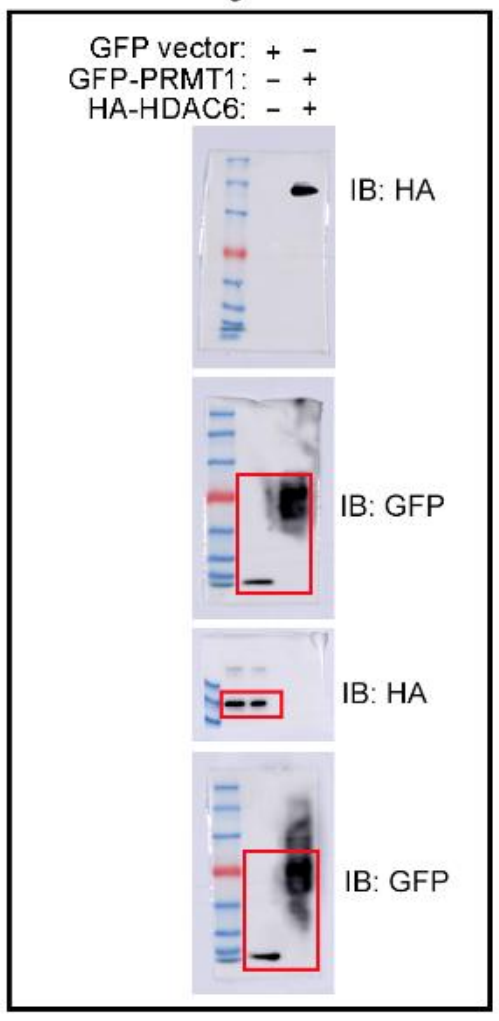

Fig. 3I

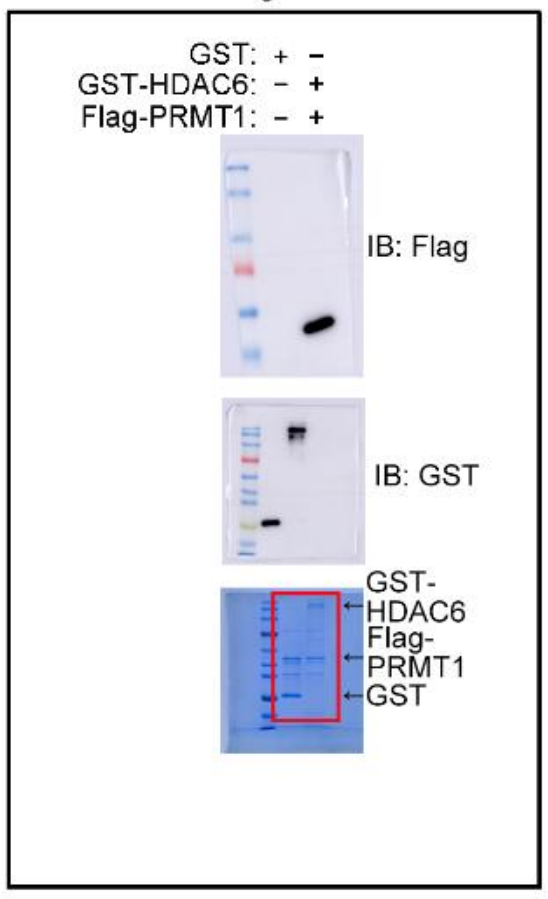

Fig. 3L

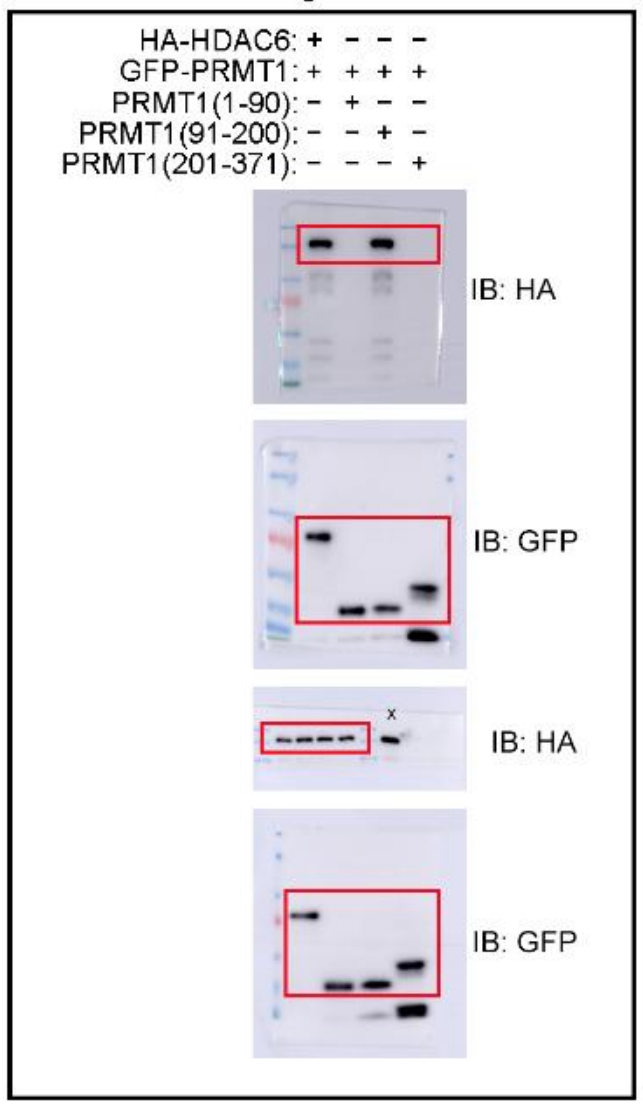

Fig. 3K

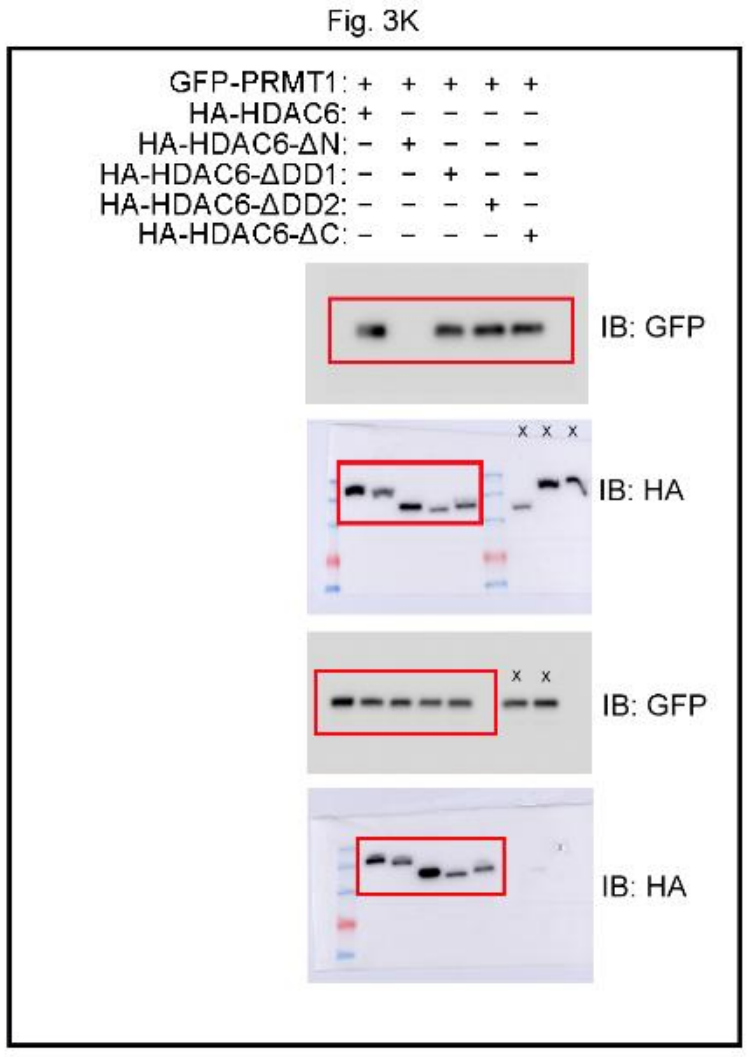

Fig. 4A

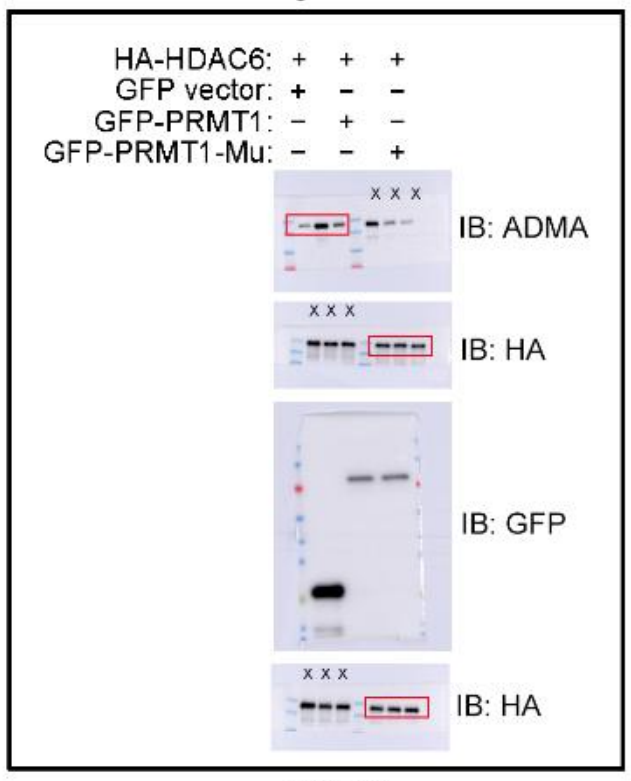

Fig. 4B

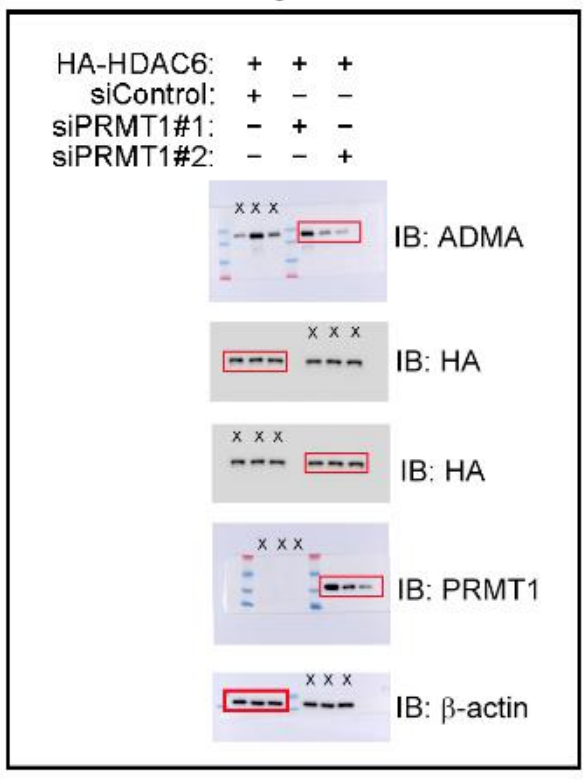

Fig. 4C

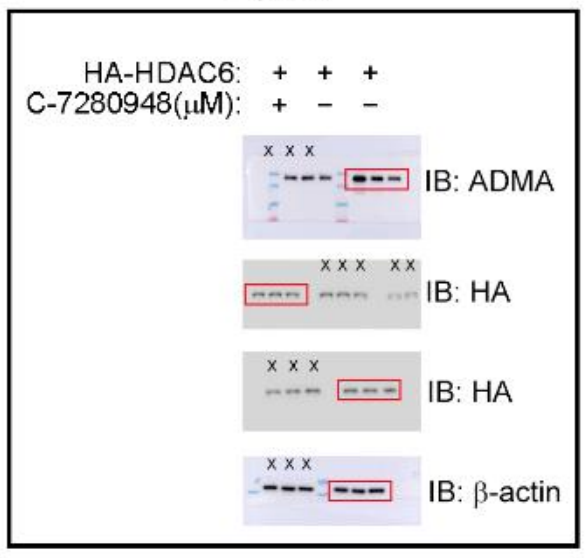

Fig. 4D

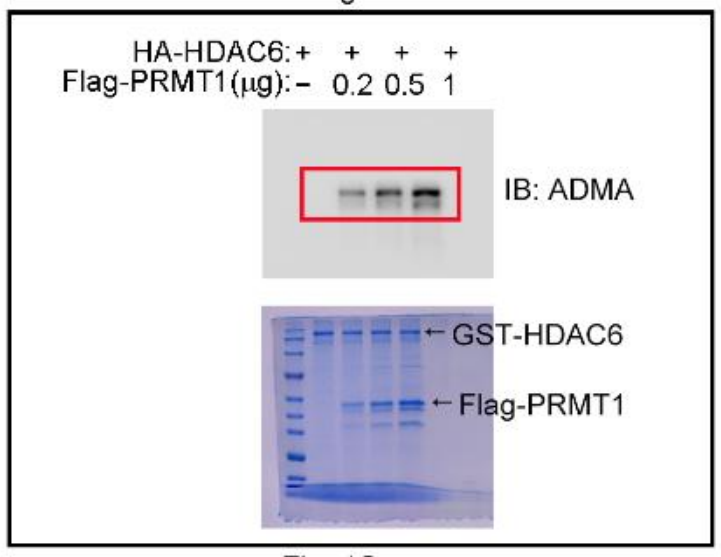

Fig. 4E

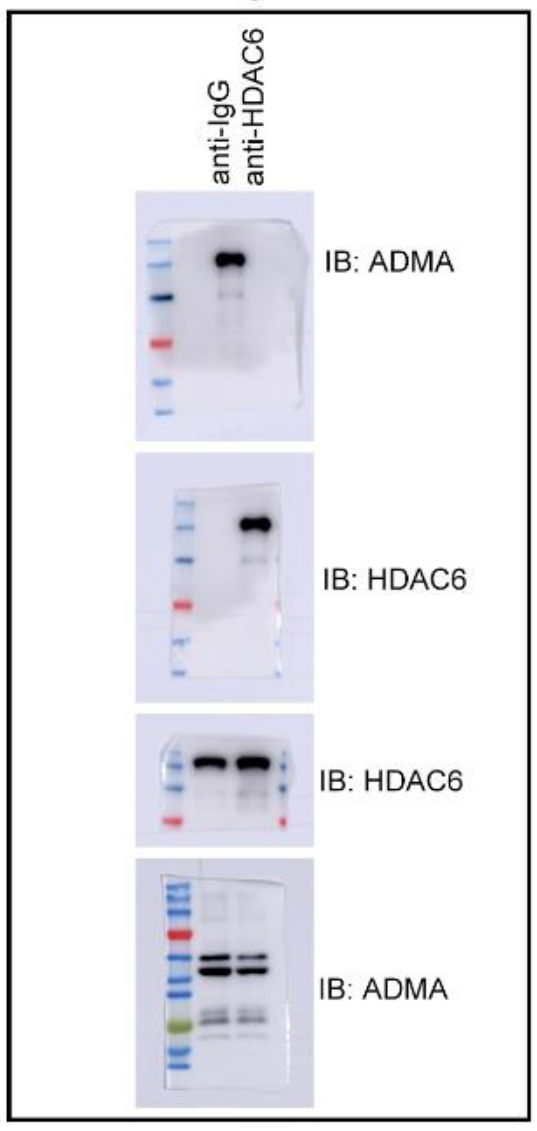

Fig. 4F

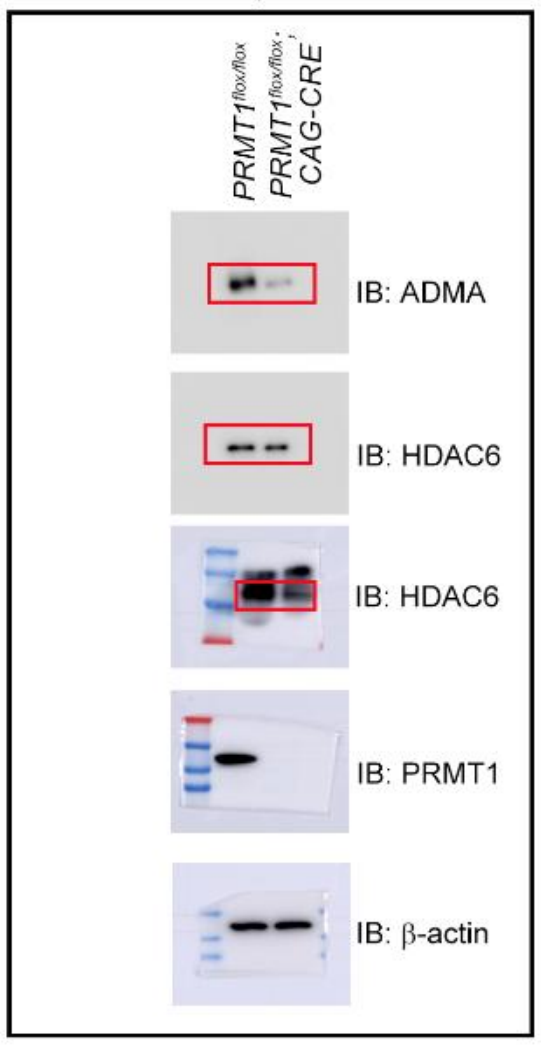

Fig. 4G

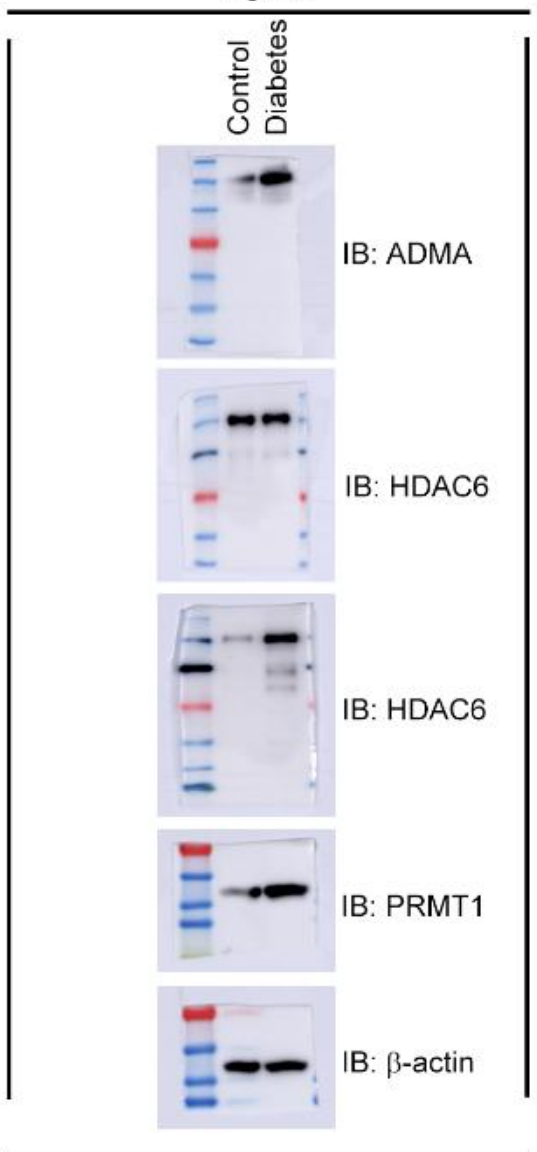

Fig. 4H

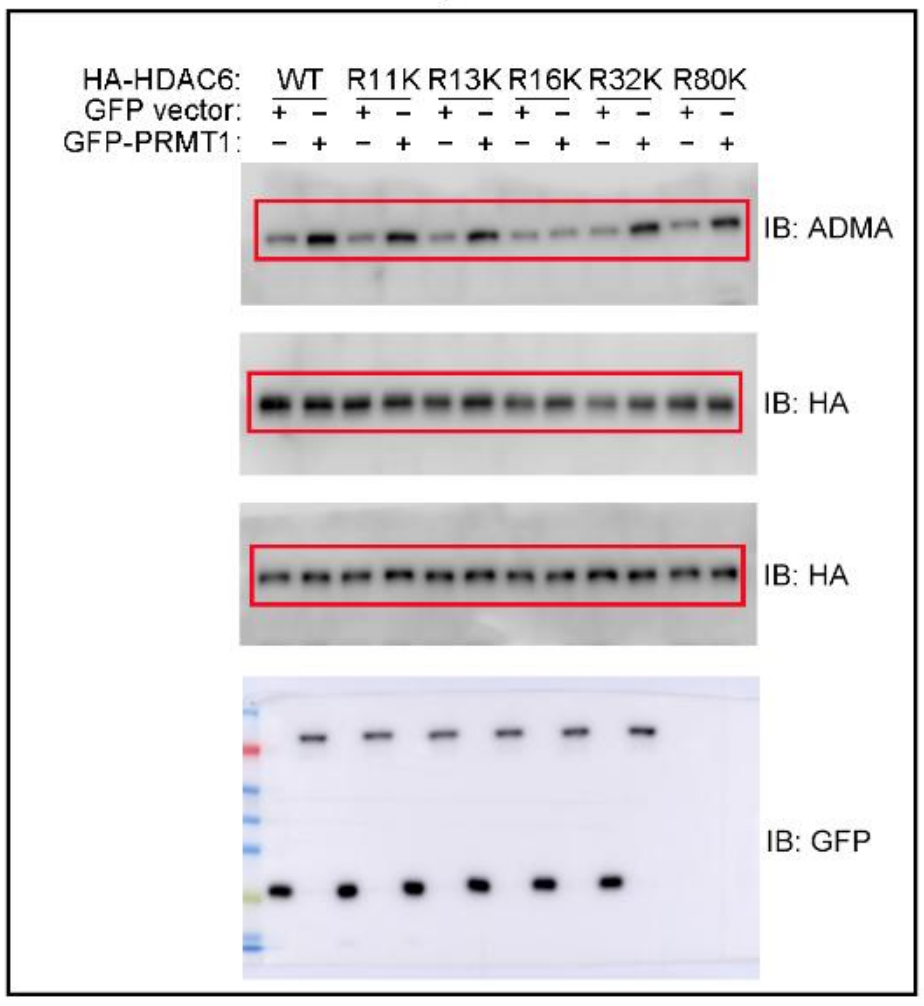

Fig. 4I

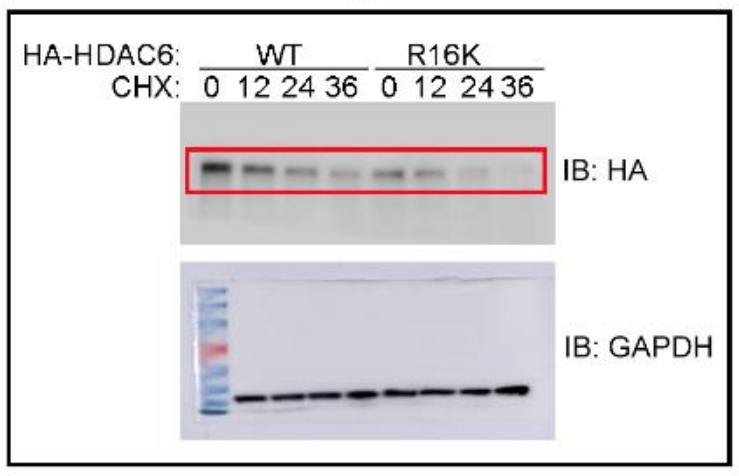

Fig. 5A

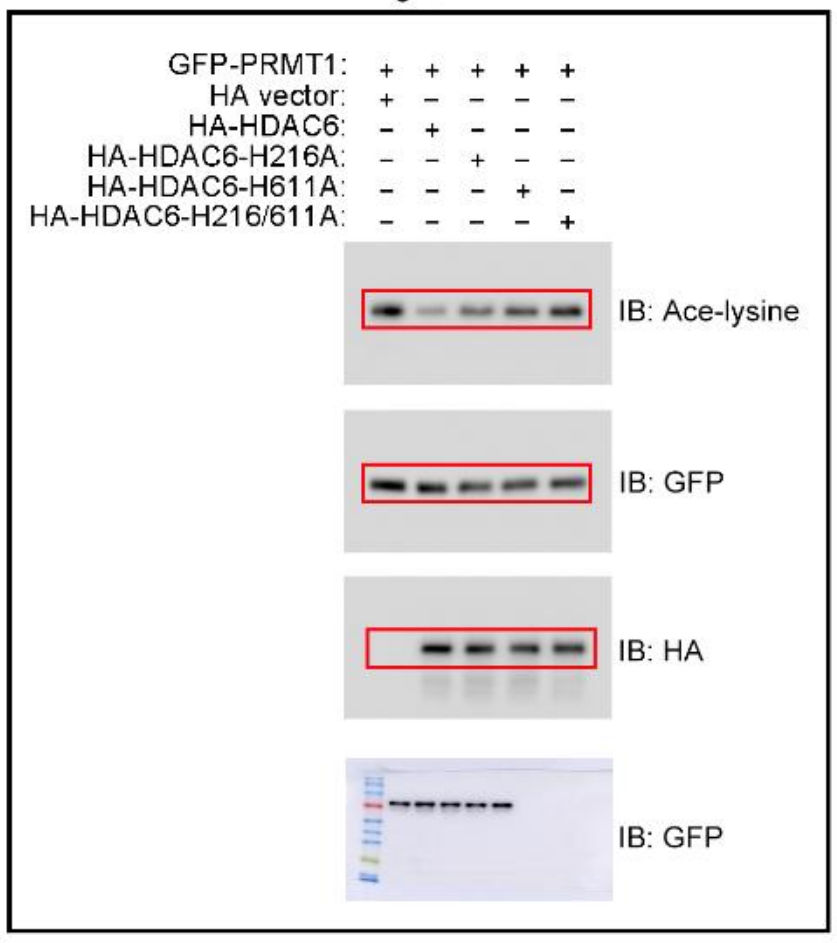

Fig. 5B

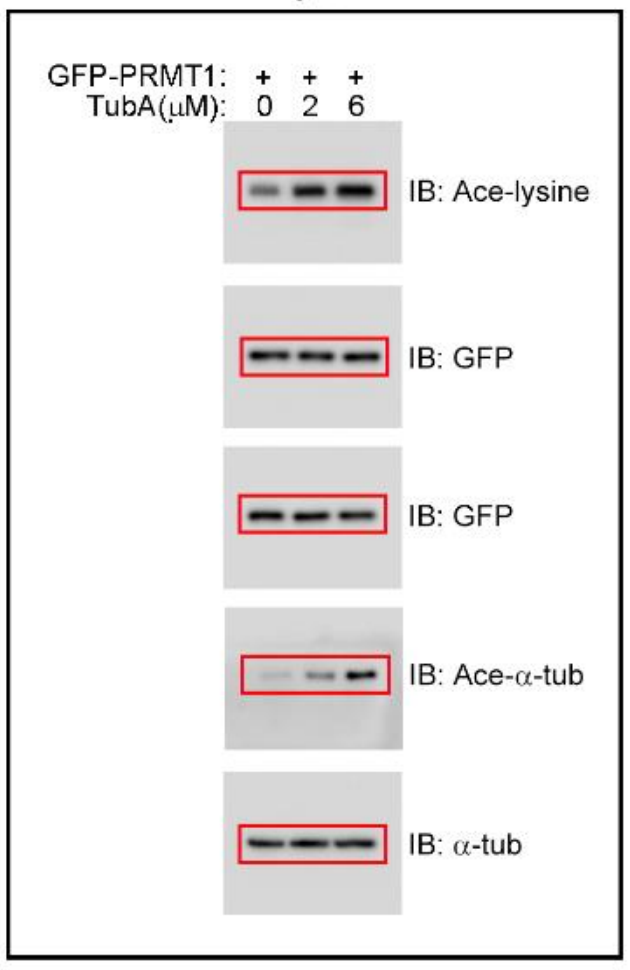

Fig. 5C

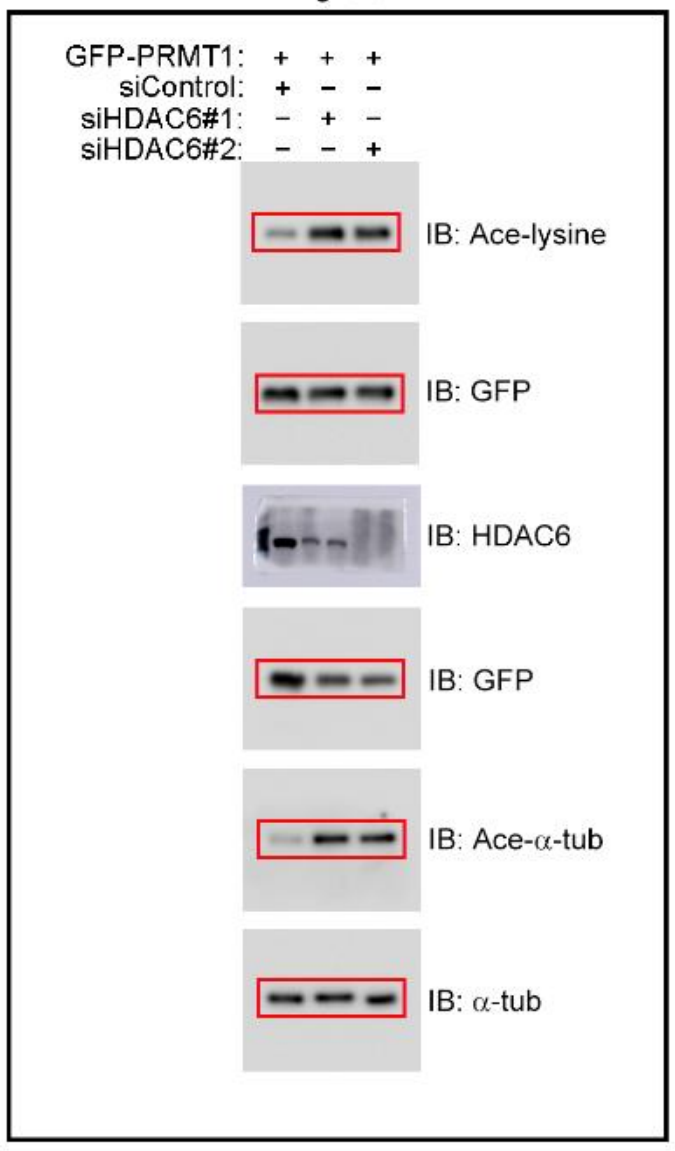

Fig. 5D

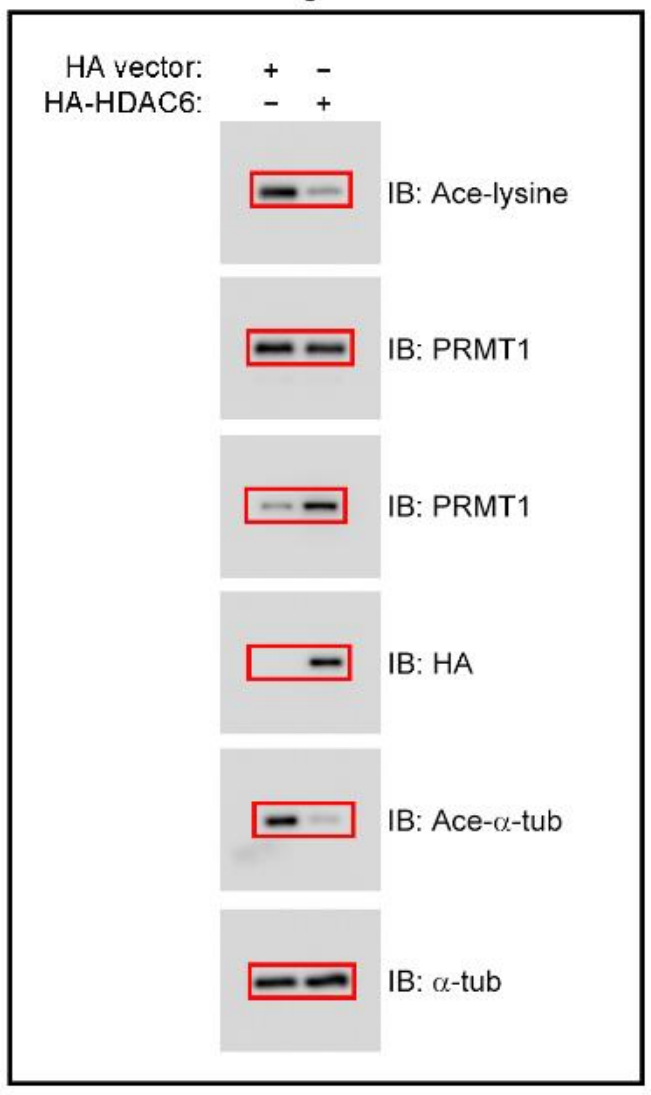

Fig. 5E

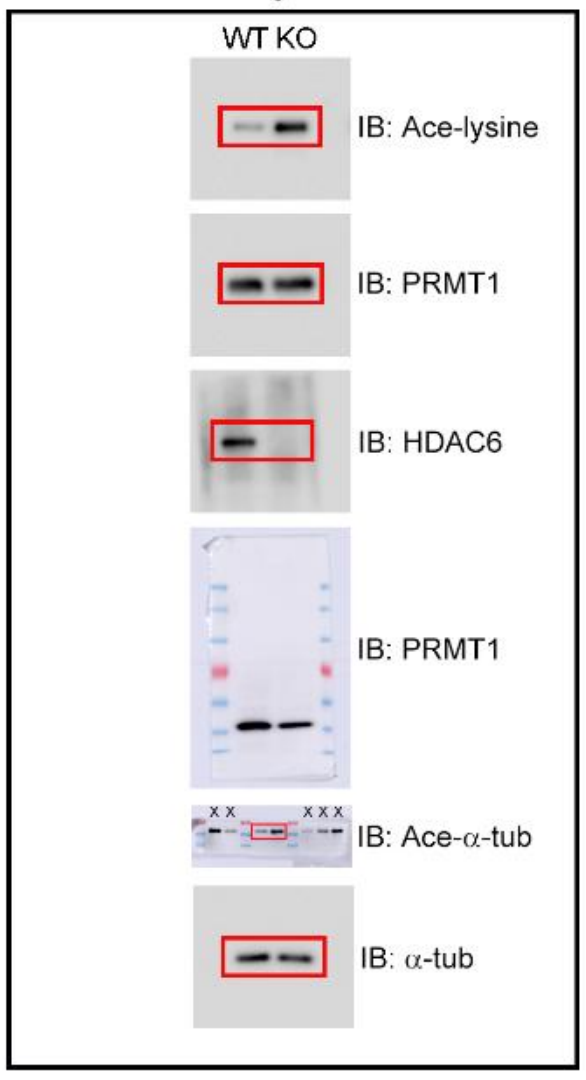

Fig. 5F

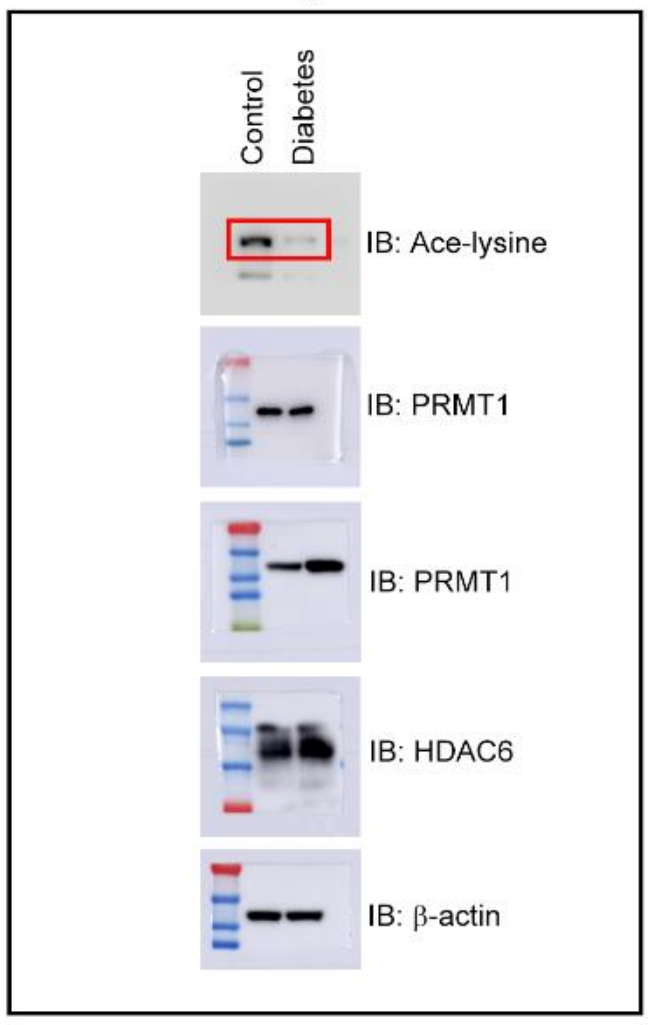

Fig. 5I

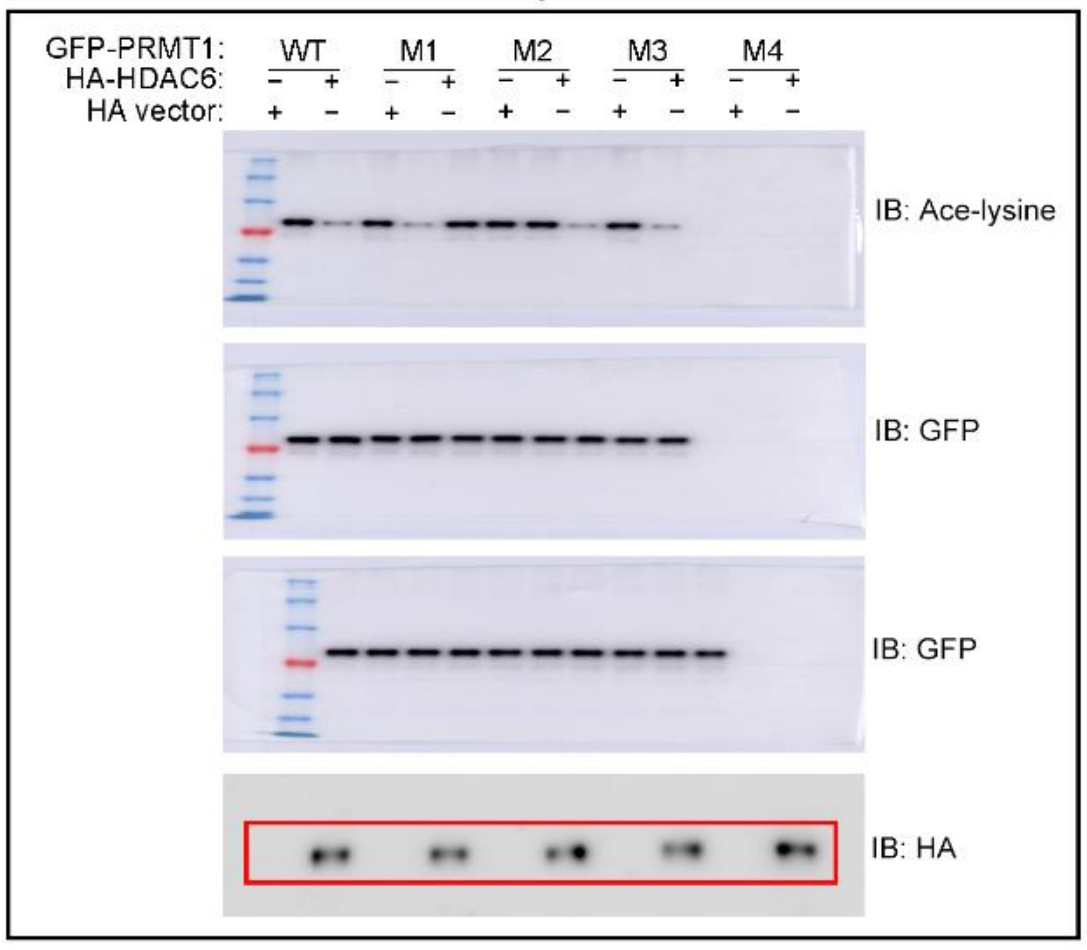

Fig. 5J

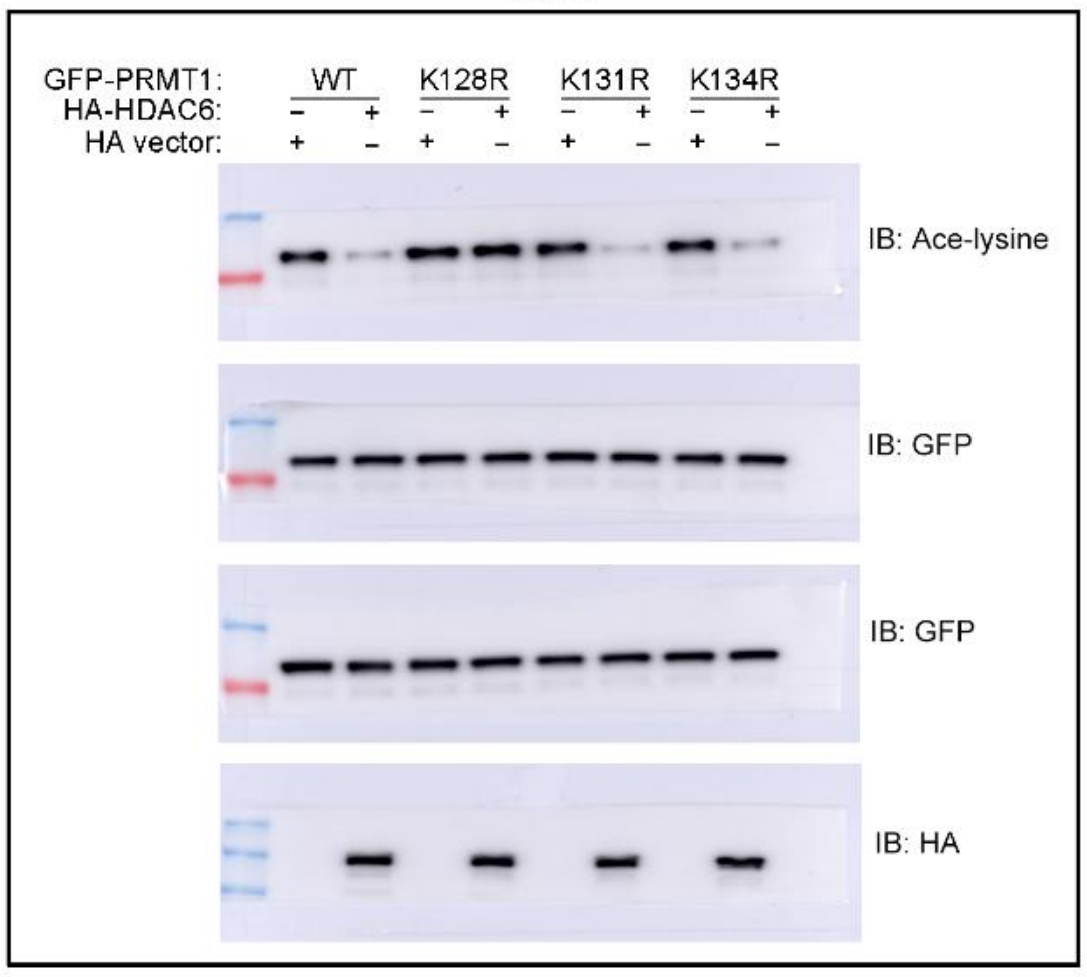

Fig. 5K

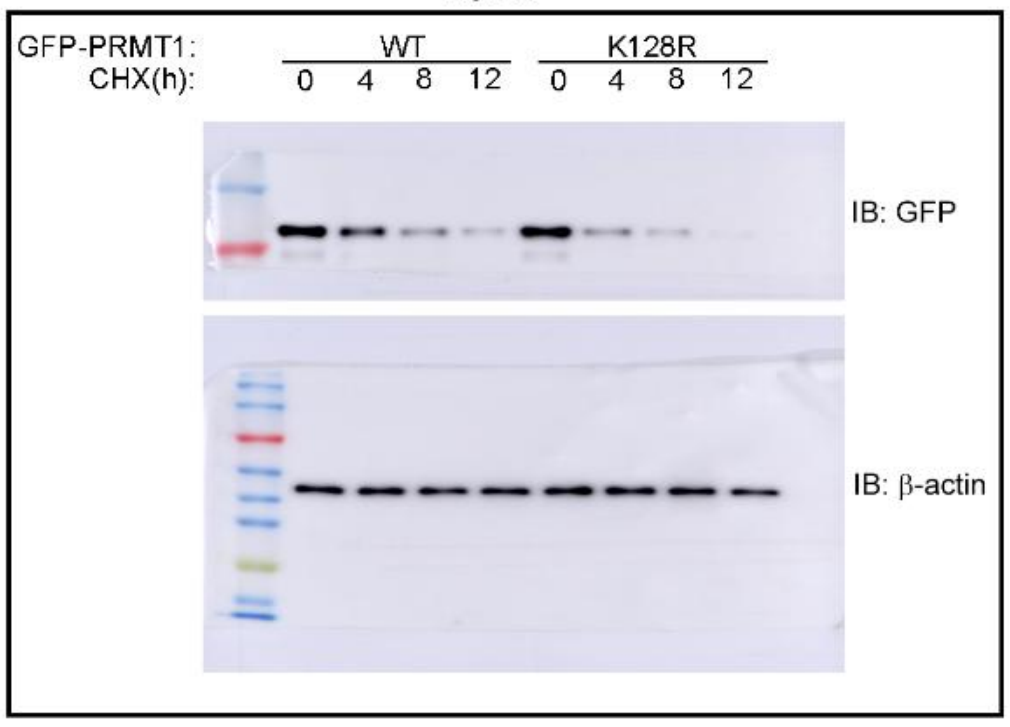

Fig. 5M

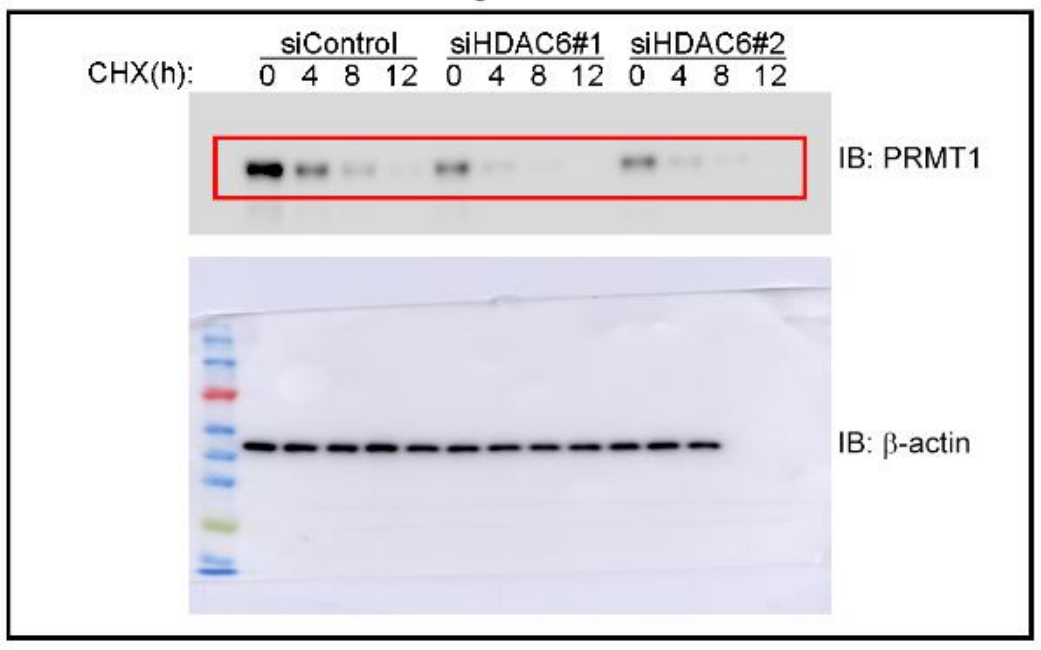

Fig. 5O

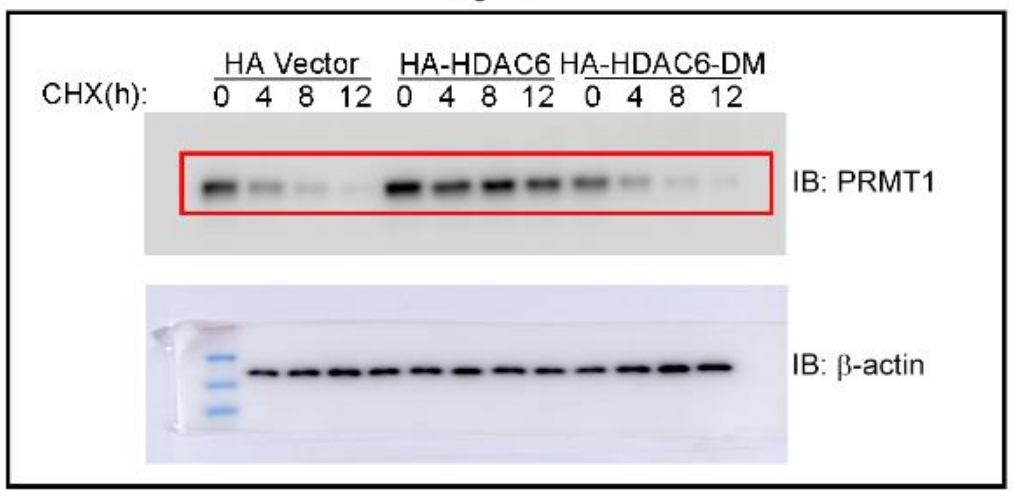

S2A Fig

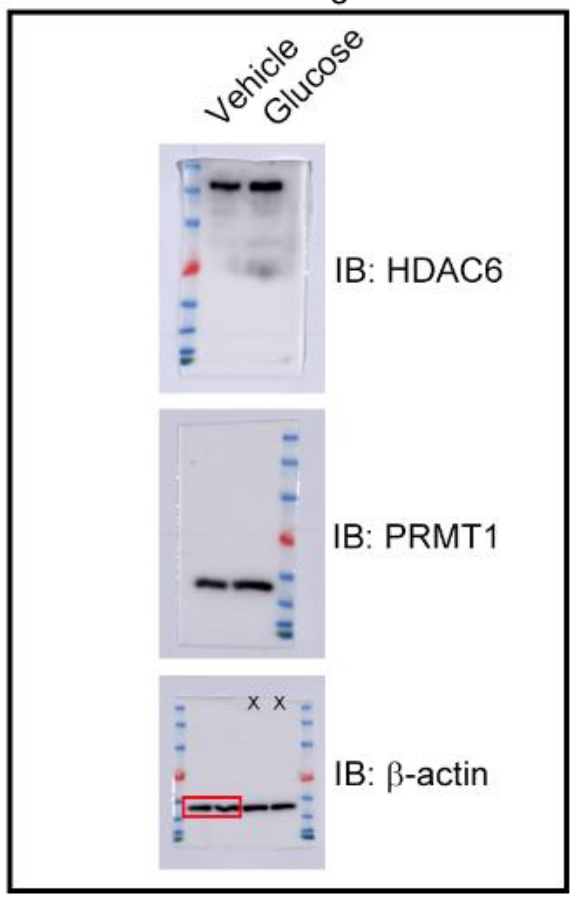

S2D Fig

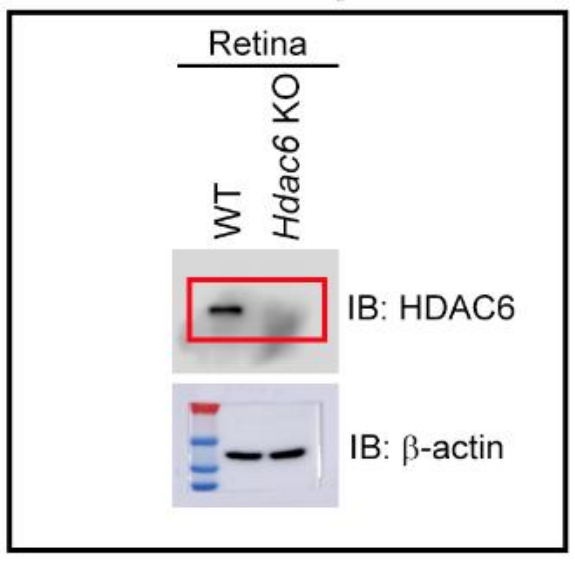

S2E Fig

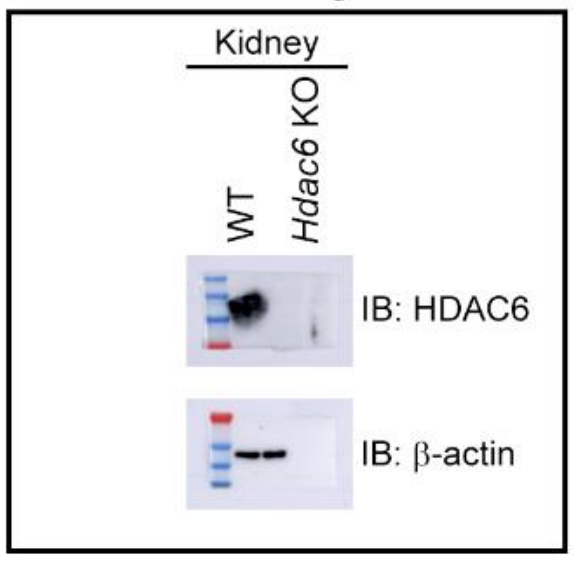

S2F Fig

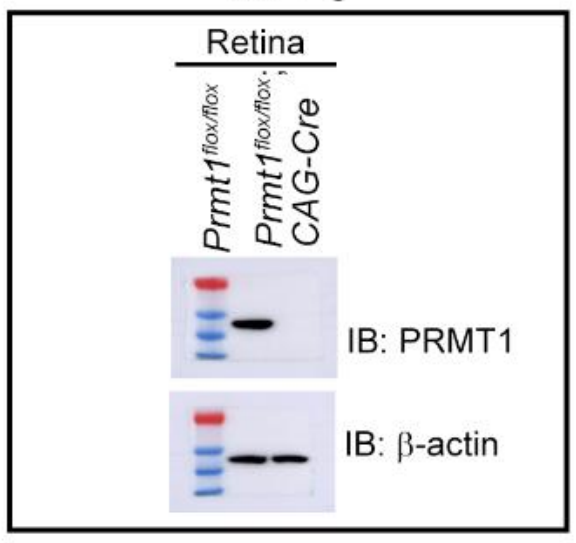

S2G Fig

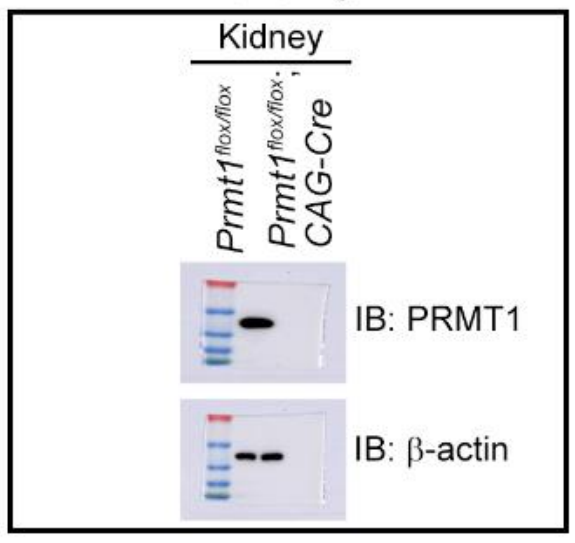

S5D Fig

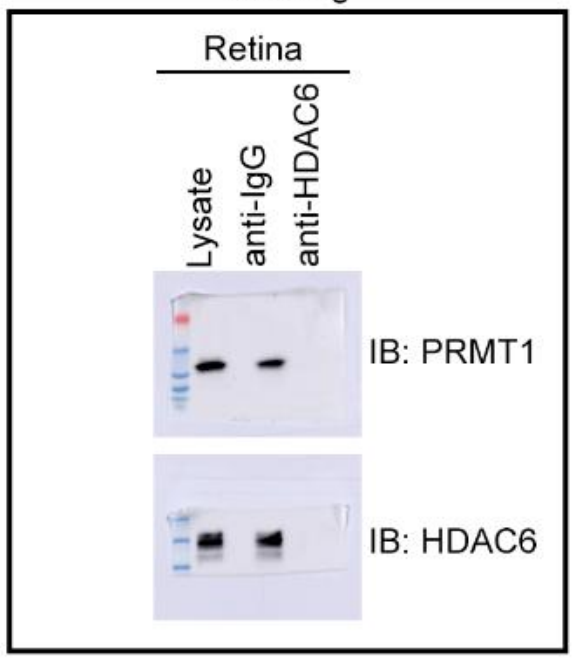

S5E Fig

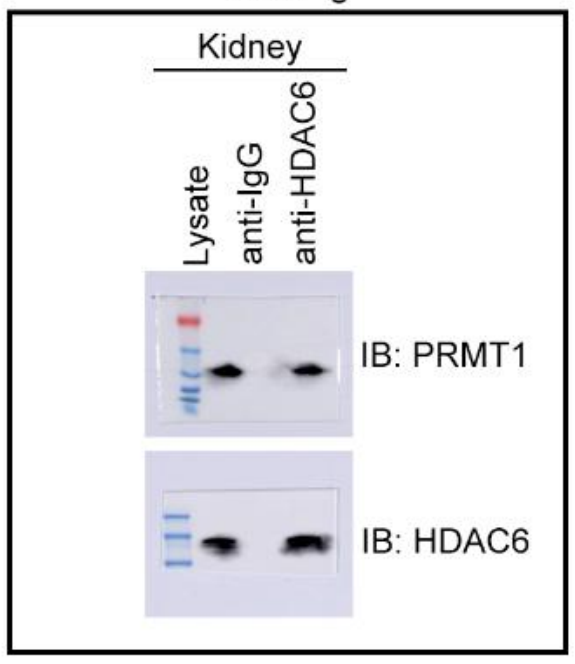

S5H Fig

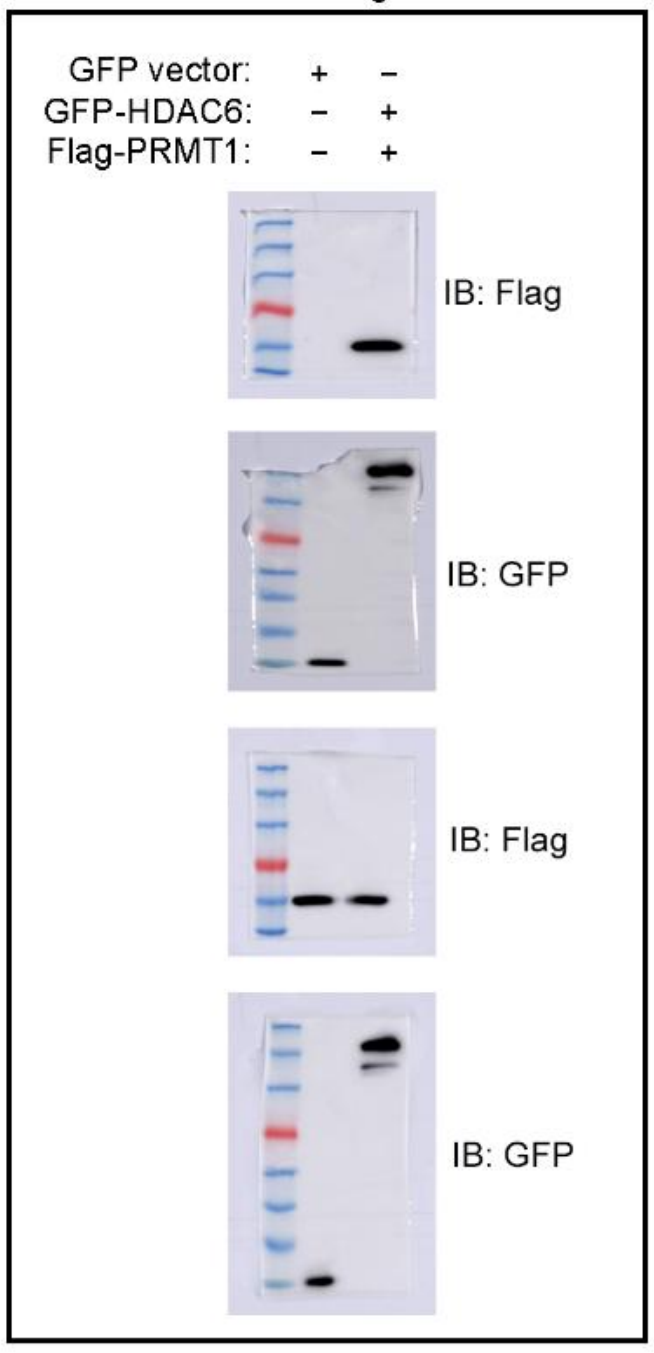

S5F Fig

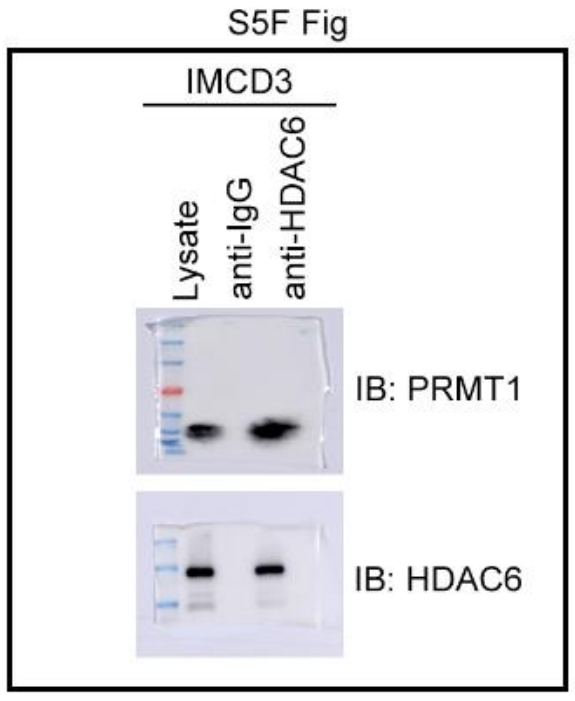

S5G Fig

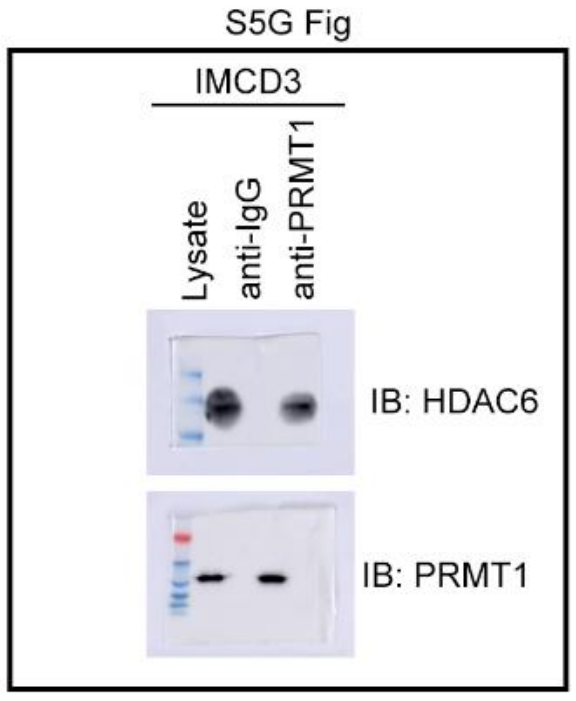

S6A Fig

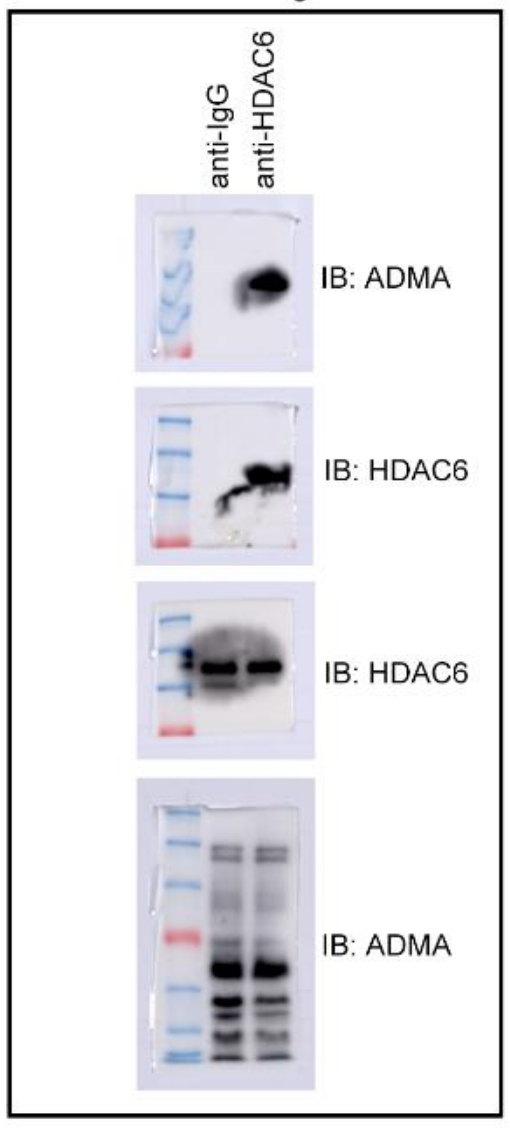

S6B Fig

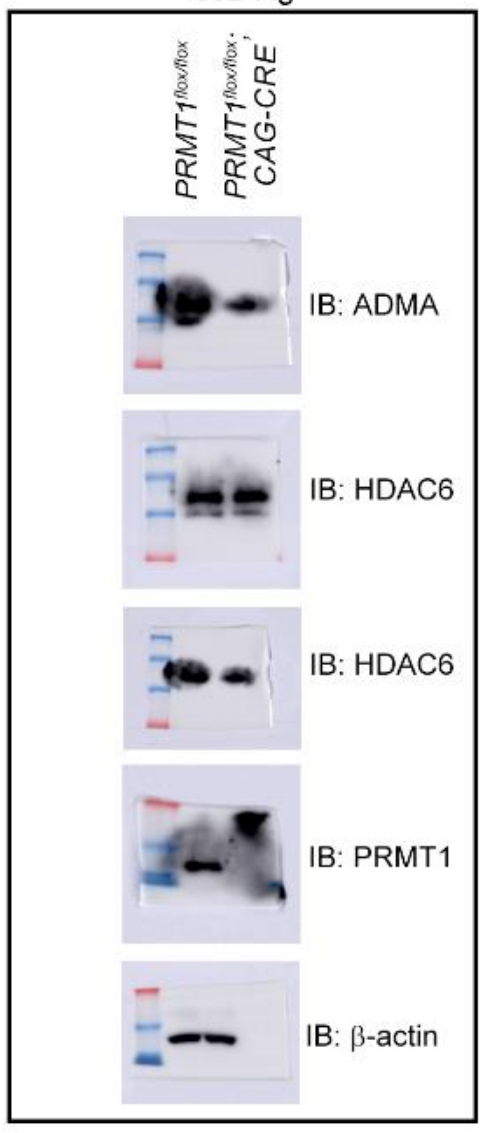

S6C Fig

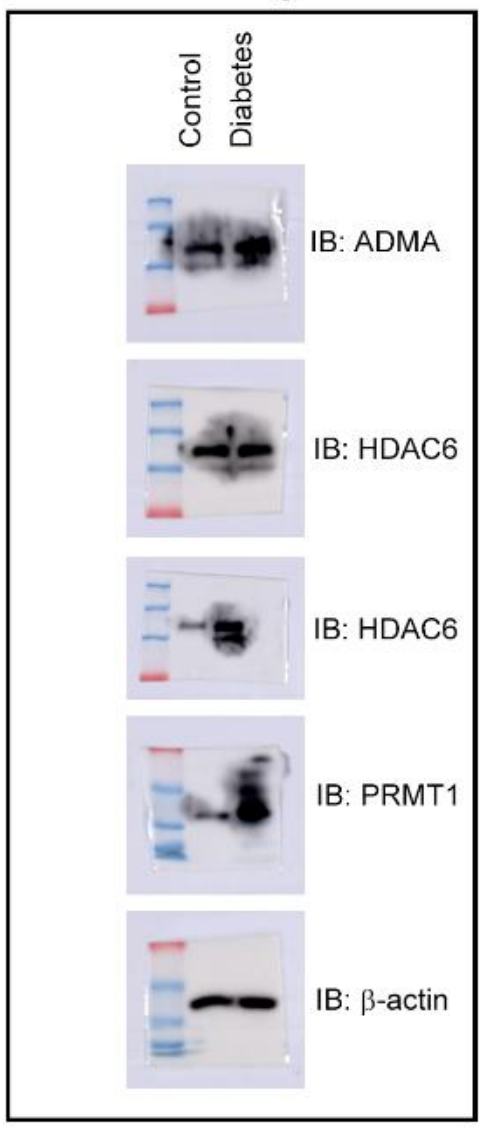

S6D Fig

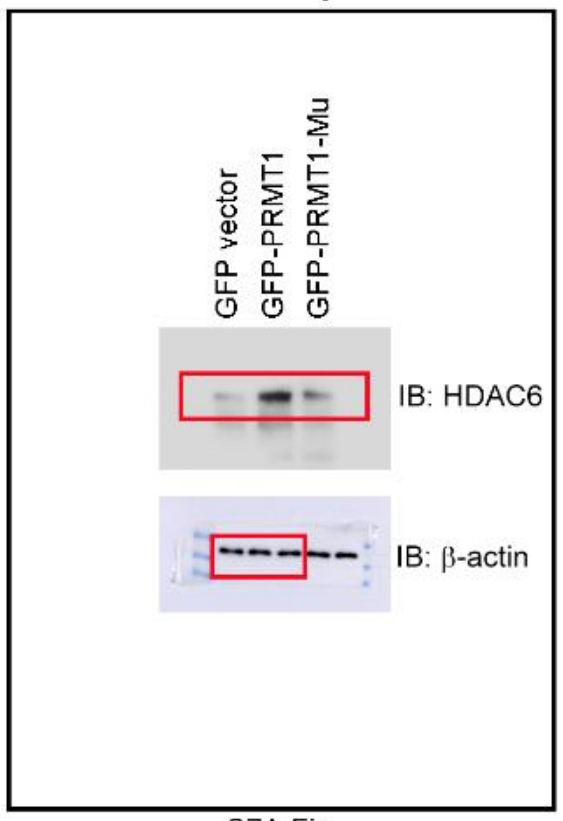

S6G Fig

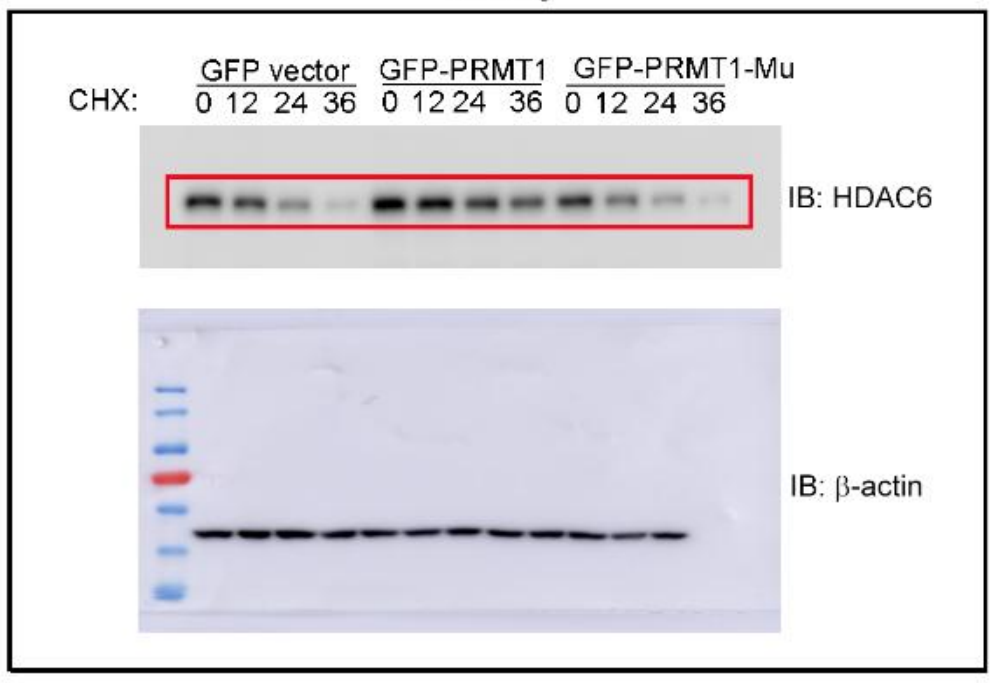

S7A Fig

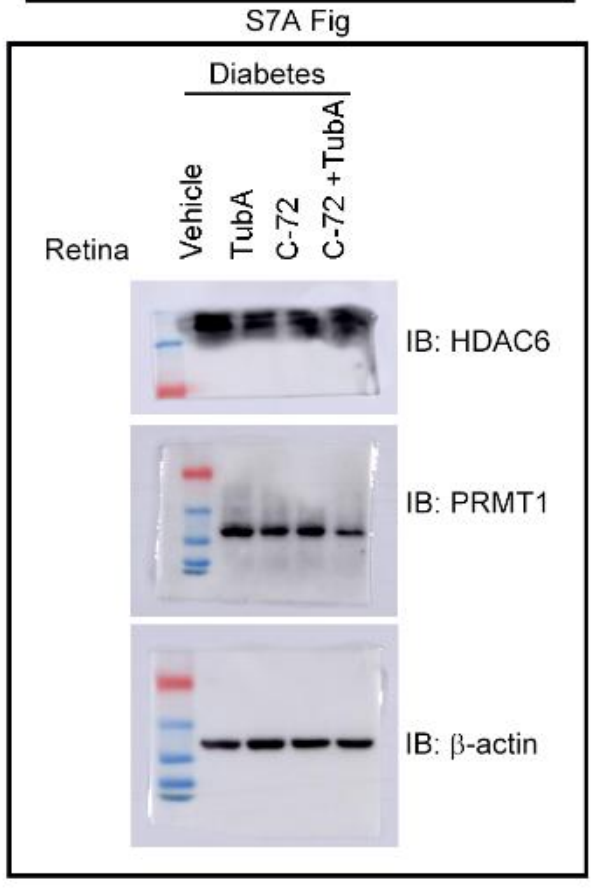

S7B Fig

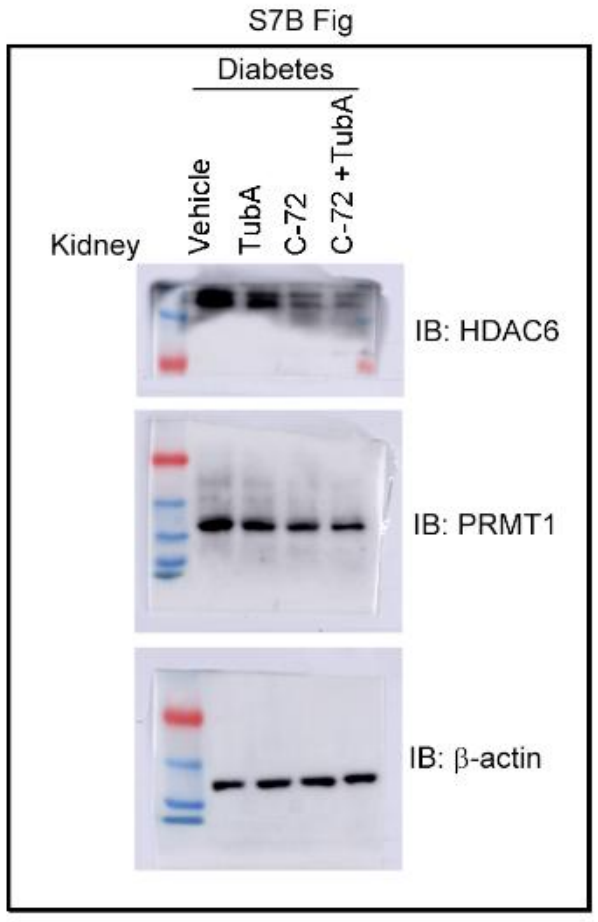

Supplement: Supplementary file 1 [file pbio.3003975.s011.pdf]
